# Toward allotetraploid cotton genome assembly: integration of a high-density molecular genetic linkage map with DNA sequence information

**DOI:** 10.1186/1471-2164-13-539

**Published:** 2012-10-09

**Authors:** Liang Zhao, Lv Yuanda, Cai Caiping, Tong Xiangchao, Chen Xiangdong, Zhang Wei, Du Hao, Guo Xiuhua, Guo Wangzhen

**Affiliations:** 1 State Key Laboratory of Crop Genetics & Germplasm Enhancement, Hybrid Cotton R & D Engineering Research Center, MOE, Nanjing Agricultural University, Nanjing, 210095, China

## Abstract

**Background:**

Cotton is the world’s most important natural textile fiber and a significant oilseed crop. Decoding cotton genomes will provide the ultimate reference and resource for research and utilization of the species. Integration of high-density genetic maps with genomic sequence information will largely accelerate the process of whole-genome assembly in cotton.

**Results:**

In this paper, we update a high-density interspecific genetic linkage map of allotetraploid cultivated cotton. An additional 1,167 marker loci have been added to our previously published map of 2,247 loci. Three new marker types, InDel (insertion-deletion) and SNP (single nucleotide polymorphism) developed from gene information, and REMAP (retrotransposon-microsatellite amplified polymorphism), were used to increase map density. The updated map consists of 3,414 loci in 26 linkage groups covering 3,667.62 cM with an average inter-locus distance of 1.08 cM. Furthermore, genome-wide sequence analysis was finished using 3,324 informative sequence-based markers and publicly-available *Gossypium* DNA sequence information. A total of 413,113 EST and 195 BAC sequences were physically anchored and clustered by 3,324 sequence-based markers. Of these, 14,243 ESTs and 188 BACs from different species of *Gossypium* were clustered and specifically anchored to the high-density genetic map. A total of 2,748 candidate unigenes from 2,111 ESTs clusters and 63 BACs were mined for functional annotation and classification. The 337 ESTs/genes related to fiber quality traits were integrated with 132 previously reported cotton fiber quality quantitative trait loci, which demonstrated the important roles in fiber quality of these genes. Higher-level sequence conservation between different cotton species and between the A- and D-subgenomes in tetraploid cotton was found, indicating a common evolutionary origin for orthologous and paralogous loci in *Gossypium*.

**Conclusion:**

This study will serve as a valuable genomic resource for tetraploid cotton genome assembly, for cloning genes related to superior agronomic traits, and for further comparative genomic analyses in *Gossypium*.

## Background

The genus *Gossypium* contains many species of great economic and scientific importance. Cotton produces the world’s most important natural textile fiber and is also a significant oilseed crop. The cotton fiber is an outstanding model in which to study plant cell elongation and cell wall and cellulose biosynthesis [1]. Genetic improvement of fiber production and processing will ensure that this natural renewable product will be competitive with petroleum-derived synthetic fibers. Moreover, modifying cottonseed for food and feed could profoundly enhance the nutrition and livelihoods of millions of people in food-challenged economies [2]. Although cotton genome sequencing has been undertaken by a scientific consortium, cotton genomics has failed to keep pace with the accomplishments in genome sequencing in other angiosperms such as *Arabidopsis thaliana*[3], poplar[4], rice [5], and grapevine[6] etc.

The genus *Gossypium* includes approximately 50 species, 45 diploid (2n = 2x = 26) and 5 tetraploids (2n = 2x = 52). Diploid cotton species contain eight genome types, denoted A-G and K [7]. Interestingly, the A genome diploids and tetraploid species produce spinnable fiber and are cultivated on a limited scale, whereas the D genome species do not [8]. In the A genome, D genome and AD genome, the genome sizes vary by approximately 3-fold, from 885 Mb in the D genome to 2,500 Mb in the tetraploid [7,9]. Genome size in cotton is not only much larger than in *Arabidopsis thaliana*, poplar, grapevine and rice, but the cotton genome has also experienced a higher frequency of genome polyploidization events than any of these species [10,11], although the grapevine genome appears to be an ancient hexaploid [6]. Much of the size variation in cotton genomes can be attributed to accumulation of transposable elements, although some lineages show evidence of specific mechanisms to remove repetitive DNA [12,13]. Repetitive elements comprise approximately 50% of the D genome [12]. Because of this, progress in cotton genome sequencing has lagged behind other flowering plants.

Genomic resources for cotton such as bacterial artificial chromosomes (BACs), expressed sequence tags (ESTs), genomic sequences, genetic linkage maps, and physical maps provide landmarks for sequence analysis and assembly. Since the first genetic map of cotton was published in 1994 [14], several high-density genetic maps composed of more than 2,000 loci have been released [15-18]. These high-density maps were constructed with multiple types of DNA markers including restriction fragment-length polymorphisms (RFLPs) [15], amplified fragment-length polymorphisms (AFLPs) [16], sequence-related amplified polymorphisms (SRAPs) [16], single nucleotide polymorphisms (SNPs) [18], and simple sequence repeats (SSRs) [16-18]. Genome-wide integration of genetic and physical maps is a prerequisite for large-scale genome sequencing, which can in turn provide initial insights into the structure, function, and evolution of plant genomes [19-21]. In the development of genomic resources in cotton, BAC libraries have been constructed for several cotton species [22-25]. The physical map of homoeologous chromosomes 12 and 26 in upland cotton [26], and a draft physical map of a D-genome cotton species (*Gossypium raimondii*) [24] have been reported.

At present, a large number of cotton sequences are publically available via the Genbank database (http://www.ncbi.nlm.nih.gov/). Of these, approximately 435,354 are expressed sequence tags (EST), including 297,214 ESTs from *G. hirsutum*, 63,577 from *G. raimondii*, 41,781 from *G. arboreum*, 32,535 from *G. barbadense*, and 247 from *G. herbaceum*. Furthermore, genome sequence information produced by several high-throughput DNA sequencing platforms, such as the Roche/454 FLX and the Illumina Genome Analyzer, have been released for several cotton species. A pilot study by the U.S. Department of Energy Joint Genome Institute (http://www.jgi.doe.gov/) to generate a whole-genome scaffold sequence for *G. raimondii* was recently completed. However, draft genome sequences lack sufficient contiguity in many genomic regions to allow for cross-species comparison of genome organization and structure [27,28]. An independent genetic map often facilitates the correct ordering of DNA segments on chromosomes and can thus clarify the changes in genome organization revealed by multiple species comparisons [29,30]. As a result, structural, functional, and evolutionary studies in *Gossypium* will largely be accelerated and a whole-genome sequence will ultimately be realized.

In this paper, we report an update to a high-density interspecific genetic map in allotetraploid cultivated cotton based on earlier work in our laboratory [16,31-34]. Using the high-density linkage map, we developed the genome-wide sequences analysis by the integration of high-density genetic map and publically-available *Gossypium* DNA sequence. This study will serve as a valuable genomic resource for tetraploid cotton genome sequencing, assembly and further comparative genomic analyses in *Gossypium*.

## Results

### A newly updated tetraploid cotton genetic map composed of 3,414 loci in 26 linkage groups

**Figure 1:**
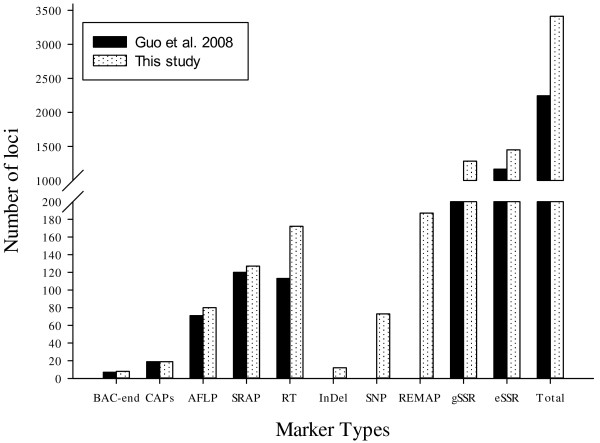
**Comparison of marker types and loci between the previously published map and the updated genetic linkage map.** Note: The InDel, SNP and REMAP markers are new to the updated map and are not included in the map of Guo et al. 2008.

**Figure 2:**
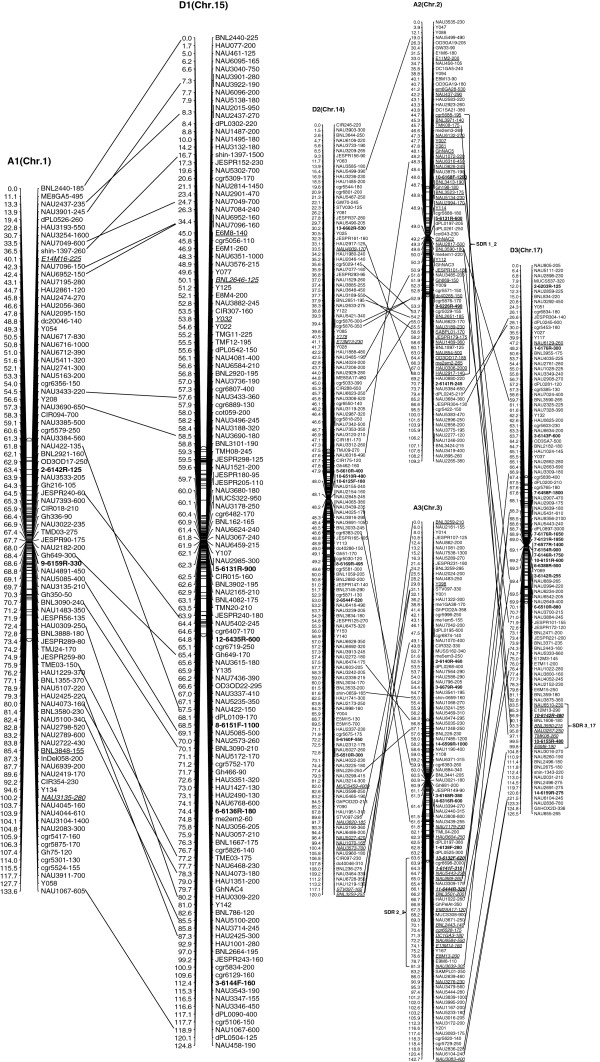
**The newly-updated genetic map for A1/D1, A2/D2 and A3/D3 homoeologous pairs.** Note: Genetic map was constructed using a BC_1_ population obtained from the interspecific cross: *G. hirsutum* L. acc. TM-1 × *G. barbadense* L. cv. Hai7124. Recombination distance is given in centimorgans (cM). Chromosomes and linkage groups are arranged by homoeologous pairs and their corresponding conventional chromosome numbers denoted in bracket. REMAP markers are shown in bold. Segregation distorted loci are underlined and italicized; loci skewed toward the heterozygote only are underlined, loci skewed toward TM-1 are both italicized and underlined. Segregation distortion regions (SDRs) are named as ‘No. SDR + linkage group’, for example, SDR1_2 refers to the first SDR (out of all SDRs) and is located on Chr. 2 linkage group.

**Figure 3:**
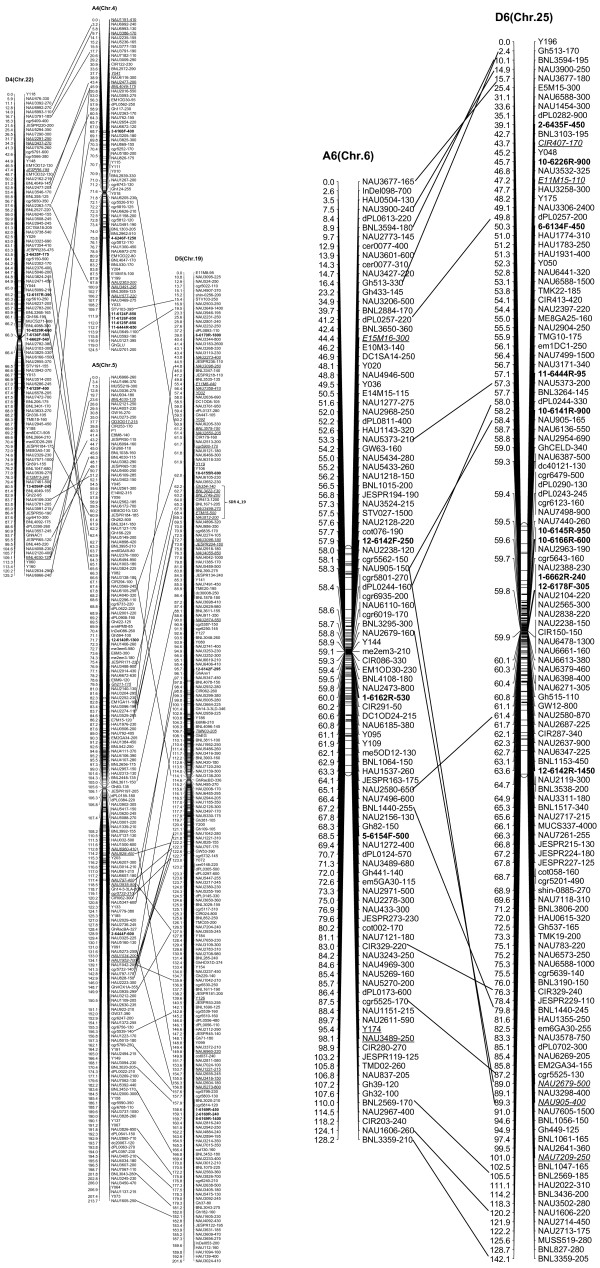
**The newly-updated genetic map for A4/D4, A5/D5 and A6/D6 homoeologous pairs**. All legends are same as described for Figure 2.

**Figure 4:**
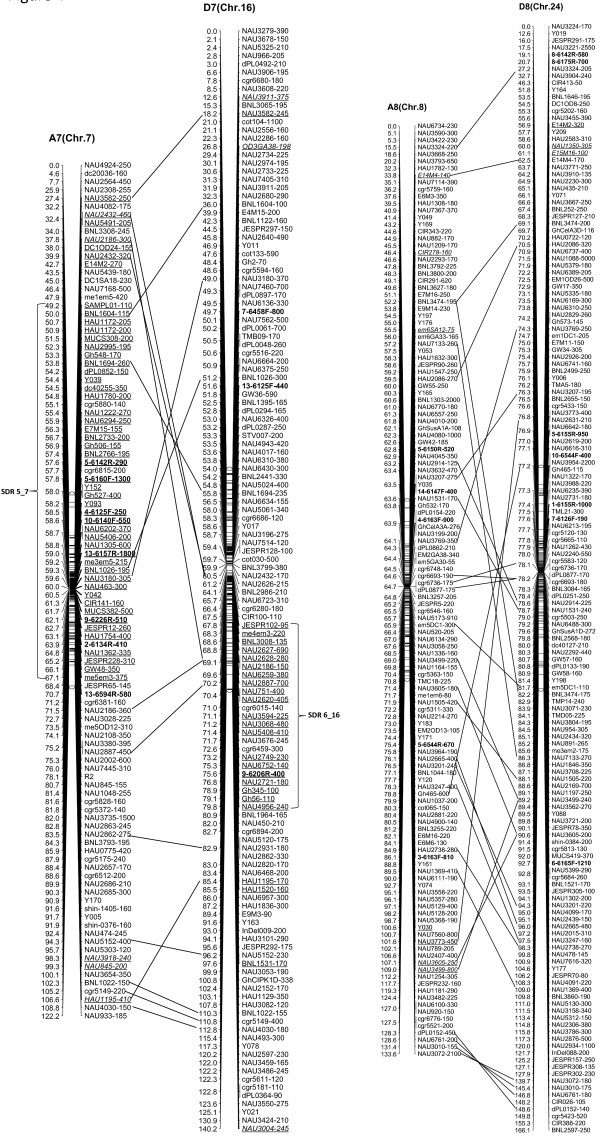
**The newly-updated genetic map for A7/D7 and A8/D8 homoeologous pairs.** All legends are same as described for Figure 2.

**Figure 5:**
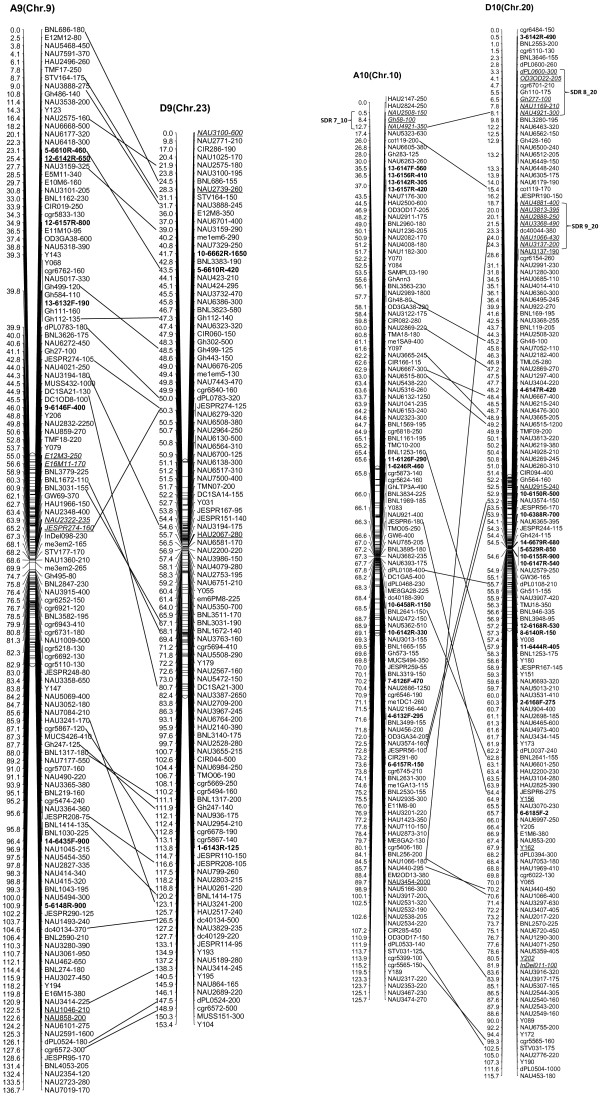
**The newly-updated genetic map for A9/D9 and A10/D10 homoeologous pairs.** All legends are same as described for Figure 2.

**Figure 6:**
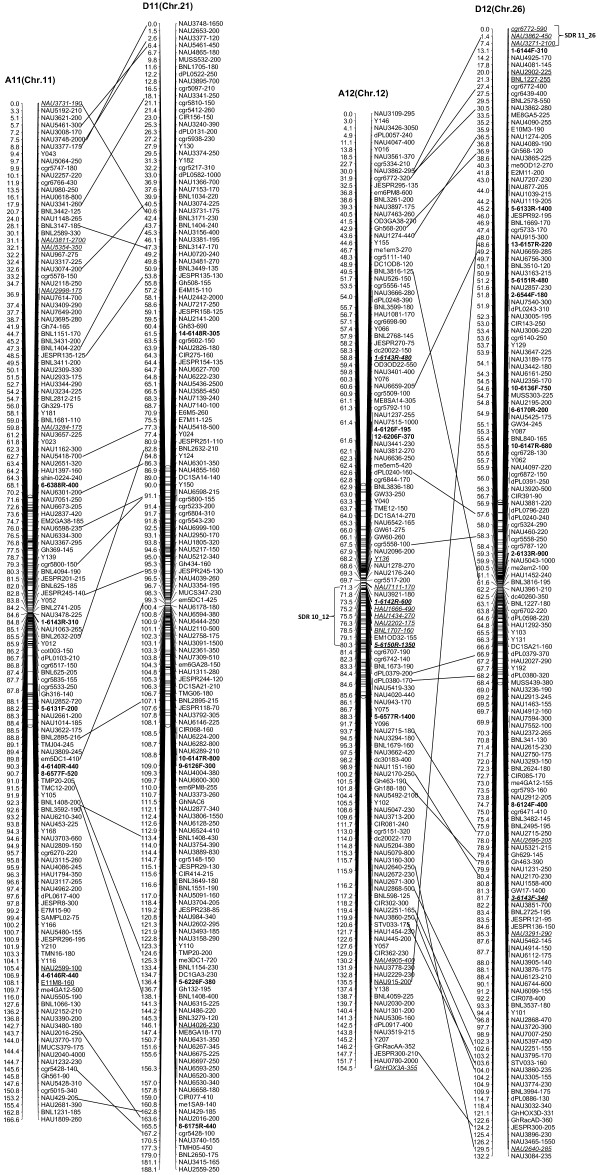
**The newly-updated genetic map for A11/D11 and A12/D12 homoeologous pairs.** All legends are same as described for Figure 2.

**Figure 7:**
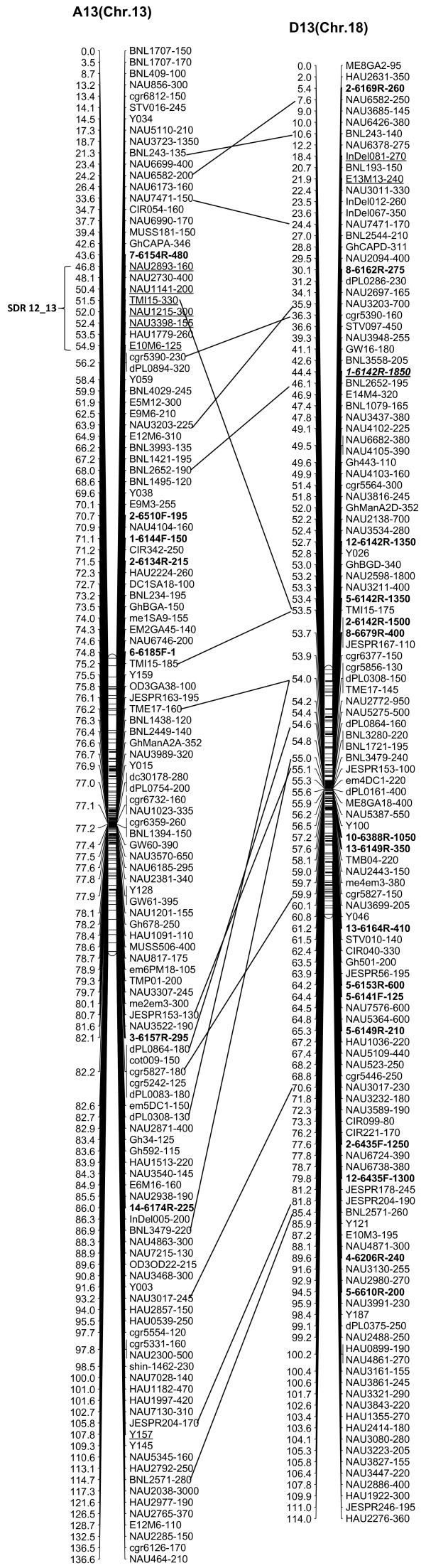
**The newly-updated genetic map for A13/D13 homoeologous pairs.** All legends are same as described for Figure 2.

We integrated an additional 1,167 polymorphic marker loci into our previously published linkage map that contained 2,247 loci and spanned 3,540.4 cM [16]. The new marker loci comprised a variety of marker types, including 534 genomic-SSR loci, 285 EST-SSRs, 187 REMAPs, 73 SNPs, 12 InDels, 59 RTs, nine AFLPs, seven SRAPs and one derived from a BAC-end sequence. Of these, three new marker types, InDel, SNP and REMAP, were used to increase the density of the new genetic map (Figure 1). As a result, we constructed a newly-updated genetic map composed of 3,414 loci in 26 linkage groups covering 3,667.62 cM with an average inter-locus distance of 1.08 cM (Figures 2, 3, 4, 5, 6, 7).

The enhanced linkage groups account for 1,559 loci (1827.6 cM) with 1.17 cM interval distance in the A-subgenome and 1,855 loci (1850.02 cM) with 1.00 cM interval distance in the D-subgenome, respectively. On average, each chromosome has 131 loci, ranging from a high of 223 loci on D5, to a low of 75 loci on A4. The longest chromosome in terms of genetic distance was A5 (Chr. 5; 213.7 cM), and the shortest was A2 (Chr. 2; 109.2 cM). Compared with the previously published map [16], intervals of >10 cM remaining in the tetraploid map were reduced to 16 - nine in the At subgenome, and seven in the Dt subgenome, with the largest number of gaps on chromosome D8 (4 gaps >10 cM) and the largest overall gap on chromosome A3 (between two adjacent loci) 22.2 cM (Additional file 1: Table S1).

### Duplication, rearrangement and translocation in allotetraploid cottons

In this new map, 693 duplicated loci were identified by 326 SSR primer pairs, with 574 duplicated, 111 triplicated, and eight tetraplicated loci (Additional file 2: Table S2). Of these, 64.07% duplicated loci sufficiently bridged 13 expected homologous At/Dt chromosomes. The remaining 249 duplicated loci were present on non-homologous chromosomes, of which 32.13% loci were found to be located on the same chromosomes, whereas 67.87% loci spanned different chromosomes. This finding implied that there had been multiple rounds of duplication and both intrachromosome and interchromosome genome rearrangements during the process of evolution (Additional file 2: Table S2). Furthermore, two post-polyploidization reciprocal translocations of A2/A3 and A4/A5 in the At subgenome were confirmed to have 27 homologous loci, with eight duplicated loci on the A3 and D2 chromosomes, seven on A2 and D3, nine on A5 and D4 and three on A4 and D5 (Figures 2, 3).

### Structure characterization of the newly updated linkage map

Of the 3,414 loci, 300 loci showed non-mendelian segregation (P < 0.05) with 137 loci skewed toward TM-1 and 163 loci skewed toward the heterozygous state (Table 1). Of these distorted loci, 180 were in the At subgenome and 120 in the Dt subgenome. These segregation-distorted loci were unevenly distributed and clustered in some regions of the 26 genetic linkage groups. A total of 12 segregation distortion regions (SDRs) were detected on 11 linkage groups. There were two SDRs in the D10 linkage group (Figure 5). Among 12 SDRs, six were on the At subgenome and six on the Dt subgenome, with 8 SDRs skewed toward *G. hirutum* TM-1 and four SDRs skewed toward the heterozygote. SDR1_2, SDR5_7 and SDR6_16 were the three biggest SDRs and they all showed distortion toward the heterozygote (Figure 2, 4).

**Table 1:** **Number and type of molecular marker loci that show segregation distortion in *Gossypium***

**Marker type**		**Total loci**	**Distorted segregation loci**	**Skewed toward**	
				**TM-1**	**Heterozygote**
SSR	eSSR	1450	140	70	70
	gSSR	1284	73	29	44
REMAP		187	21	9	12
RT		172	22	6	16
SRAP		127	13	5	8
AFLP		80	24	14	10
SNP		73	4	3	1
CAPs		19	0	0	0
InDel		12	2	1	1
BAC-end		8	1	0	1
morphology		2	0	0	0
Total		3414	300	137	163

The 3,414 loci were not evenly distributed on the cotton chromosomes, with more loci on the Dt-subgenome than the At-subgenome. To better understand the locus distribution on each chromosome, we analyzed the frequency of loci along 10 centiMorgan (cM) bins on the linkage map (Additional file 3: Figure S1). Most chromosomes had a similar marker density distribution, with the highest peak located near the center of linkage groups; furthermore, the A9, D5, and D9 linkage group each had two main peaks. The regions of high marker density in each chromosome indicated the presence of recombination suppression in these regions, which might be related to the heterochromatic regions [35,36]; the main peaks on the each chromosome should cover the centromeric areas.

Clusters of loci were also observed in 26 linkage groups, of which 86 clusters involved in 617 loci (≥5 loci/cM) that were discovered in 25 linkage groups besides A1 (Chr. 1). Of these, 31 clusters contained 229 loci from the At subgenome, and 55 clusters contained 388 loci from the Dt subgenome. Nineteen candidate gene islands (≥5 EST-SSR loci/cM) and one retrotransposon-rich region were discovered by cluster distribution of marker loci. For example, the cluster that spanned from 106.83 cM to 107.40 cM on A5 (Chr. 5) contained six EST-SSR markers that should have been a gene-rich region. The cluster composed of 15 loci (8 loci from REMAP makers) on D3 (Chr. 17) may be a retrotransposon-rich region (Additional file 4: Table S3).

Of 3,414 loci, with the exception of AFLP and SRAP, 3,324 (97.36%) were from informative sequence-based markers. These highly informative sequence-based markers will be suitable for aligning the sequence information to linkage groups and finishing the integration between the DNA sequences and the high-density genetic map in cotton.

### Integrating the genetic map with cotton DNA sequence resources

A total of 413,113 ESTs and 195 BACs from four major cotton species were extracted from the NCBI GenBank database as a sequence reference pool, and 3,324 sequence-based markers were used as probes to anchor and cluster these physical EST and BAC sequences by a highly specific *in silico* PCR-based method. As a result, 2,111 primer pairs (63.5%) successfully amplified DNA fragments from 14,243 ESTs and 63 BACs (Additional file 5: Table S4). At the same time, the chromosomal locations of 63 BACs were further confirmed by combining PCR-based *in silico* anchor results with PCR experimental amplification analysis according to the criteria described in the previous study [16,37]. The remaining 1,213 primer pairs (36.5%) did not amplify any DNA products from the reference pool.

Based on PCR *in silico* anchor results, we found that 11 BACs were anchored on both A- and D-genome homoelogous chromosomes; 2,111 primer pairs amplified *in silico* DNA sequences from four cotton species, with 762 primer pairs directing amplification of same-sized DNA fragments, and 1,349 primer pairs giving PCR products with different predicted sizes. These results showed higher-level sequence conservation and molecular size differences in orthologous and paralogous loci in the natural evolutionary process of different *Gossypium* species.

To identify the genes corresponding to 14,243 ESTs and 63 BACs, 2,111 clusters were grouped and each EST cluster was assembled into the longest possible unigene. For 63 BACs anchored to corresponding subgenome chromosomes, all genes in each BAC were predicted based on *ab initio* method using the Fgenesh program for further functional analysis. In total, 2,748 candidate genes were mined for subsequent bioinformatics analysis (Additional file 5: Table S4).

### Functional annotation based on Gene Ontology

All candidate unigenes were subjected to homology analysis against NCBI the RefSeq plant protein database to obtain putative functional annotations using Blastx with a cut-off E value set to 10^-5^. Of 2,748 unigenes, 2,258 sequences (82.17%) had homology to protein sequences. Among these, 1,901 were matched known proteins, and 357 were unknown or hypothetical proteins (Additional file 6: Figure S2). The remaining 490 sequences had no homologs in the plant Refseq database and could be either 3′ or 5′ untranslated regions (UTRs) of genes with very short coding regions, or they could represent novel genes [38].

The E value distribution of the top-hits in the RefSeq database showed that 88.81% of the mapped sequences had high homology (<1e-20), whereas 11.19% ranged from 1e-07 to 1e-20 (Additional file 7: Figure S3A). Likewise, the similarity distribution showed that 92.82% of the sequences had a similarity higher than 50%, while 7.18% of the hits had a similarity ranging from 35.9% to 50% (Additional file 7: Figure S3B). Through top-hit species distribution statistics, the majority of sequences were matched to the *Ricinus communis* genome (26.57%), followed by *Populus trichocarpa* (25.24%), *Vitis vinifera* (23.03%) and *Glycine max* (12.18%), which showed that a closer genetic relationship existed between them (Additional file 7: Figure S3C).

### Functional classification and pathway analysis

Of 2,748 unigenes, 1,890 (68.78%) were mapped to the GO hierarchy with characterized biochemical and physiological functions involving biological processes, molecular functions, and cellular components (Additional file 8: Table S5). At a secondary level, the majority of the GO terms were grouped into cellular process (27.15%) and metabolic process (25.81%) categories within biological processes, binding (50.56%) and catalytic activity (40.20%) categories within molecular functions, and cell (56.13%), and organelle (34.76%) categories within cellular components (Additional file 9: Figure S4).

A comparison with the Kyoto Encyclopedia of Genes and Genomes database (KEGG) showed that the metabolic-related enzymes encoded by 582 unigenes were located in metabolic maps based on the KEGG pathway classification (Additional file 10: Table S6). Most of the sequences localized to the metabolism category (94.85%), followed by organismal systems (3.26%), followed by the categories of environmental information processing (1.37%), and genetic information processing (0.52%); the categories of cellular processes and human diseases were not found. In the category of metabolism, the mapped enzymes were mostly involved in carbohydrate metabolism (146 genes), amino acid metabolism (82 genes), and energy metabolism (66 genes). In the category of organismal systems, all 19 mapped genes were attributed to the plant immune system classification. Only eight genes were involved in signal transduction of environmental information processing (Additional file 11: Figure S5).

### Integrating ESTs/genes with previously reported quantitative trait loci (QTL) related to fiber quality

Of 2,748 candidate genes, 2,111 were mainly from the ESTs of developing fibers in *Gossypium*. To further confirm the potential function of these genes in the fiber development process, integration analysis was performed between the ESTs/genes and previously reported cotton fiber quality quantitative trait loci (QTL) [39-53]. As a result, 337 ESTs/genes related to fiber quality traits were integrated with 132 previously reported cotton fiber QTL. All integrated fiber quality QTL intervals had at least one EST/gene, and some had several (Table 2), which indicated the important roles in fiber quality of these genes. Overall, 132 QTL were not randomly distributed across chromosomes, with 35 on the At subgenome involving 100 fiber quality-related ESTs/genes and 97 on the Dt subgenome involving 237 fiber quality-related ESTs/genes. This indicated important ESTs/genes related to fiber quality existed in the Dt subgenome in tetraploid cotton. On the D8 chromosome, 48 QTL associated with elite fiber quality were clustered in the chromosome region within a 40-cM interval; meanwhile, 60 fiber quality-related ESTs/genes were also detected in this region. A meta-analysis was further performed using BioMercator software, both two QTL clusters simultaneously related to several fiber quality traits and the corresponding ESTs/genes involved in these QTL clusters were detected. Some important genes, responsible for cotton fiber quality traits reported previously [54], were found in the two QTL clusters region. For example, genes encoding cellulose synthase catalytic subunit and vacuolar h + −translocating inorganic pyrophosphatase were found in the first QTL cluster region, and genes encoding fasciclin-like arabinogalactan protein, sucrose synthase, and pectin acetyl esterase family protein were located in the second QTL cluster (Additional file 12: Table S7). This result indicated that these enriched ESTs/genes in these regions were important for improving cotton fiber quality, and should be studied in depth regarding their molecular function.

**Table 2:** Integration analysis of ESTs/genes with previously reported quantitative trait loci (QTL) related to fiber quality*

**Chromosome**	**qFE**	**qFL**	**qFF**	**qFS**	**qFU**	**QTL Total**	**ESTs/genes**
A1(Chr.1)	1	0	1	0	1	3	4
A2(Chr.2)	0	1	0	0	0	1	2
A3(Chr.3)	0	1	0	0	0	1	1
A4(Chr.4)	0	0	0	0	0	0	0
A5(Chr.5)	0	0	0	0	1	1	2
A6(Chr.6)	1	0	1	0	1	3	3
A7(Chr.7)	0	0	0	0	0	0	0
A8(Chr.8)	3	0	3	3	1	10	40
A9(Chr.9)	0	4	1	2	1	8	11
A10(Chr.10)	0	0	0	0	0	0	0
A11(Chr.11)	3	1	0	0	1	5	26
A12(Chr.12)	0	0	0	0	1	1	6
A13(Chr.13)	1	1	0	0	0	2	5
D1(Chr.15)	0	0	0	0	1	1	6
D2(Chr.14)	1	1	0	0	0	2	20
D3(Chr.17)	0	1	2	0	1	4	28
D4(Chr.22)	1	0	0	0	0	1	2
D5(Chr.19)	0	1	0	0	0	1	15
D6(Chr.25)	3	0	2	0	0	5	11
D7(Chr.16)	1	0	3	4	0	8	34
D8(Chr.24)	10	13	9	13	3	48	60
D9(Chr.23)	1	3	1	1	3	9	12
D10(Chr.20)	1	0	1	1	1	4	11
D11(Chr.21)	1	0	1	3	1	6	8
D12(Chr.26)	0	1	1	1	0	3	10
D13(Chr.18)	1	1	3	0	0	5	21
**At**	9	8	6	5	7	35	100
**Dt**	20	21	23	23	10	97	237
**Total**	29	29	29	28	17	**132**	**337**

* The fiber quality traits mainly included fiber length (FL), fiber strength (FS), fiber fineness (FF), fiber elongation (FE), and fiber uniformity (FU). QTL related to fiber quality traits were reported previously from references [39-53].

## Discussion

### A high-density genetic map is an important tool in cotton genomics research

High-density genetic maps have become an indispensible resource for elucidating genome structure, function and evolution, and are particularly important in polyploidy crops such as potato, cotton and wheat [7,10,55,56]. As the field of cotton structural genomics develops, the high-density genetic map will provide many important opportunities for mining information from important genes and QTL, implementing the integration of the genetic map with the physical map, and further building a solid foundation for cotton genome assembly and utilization.

In the present study, a high-density genetic map comprising 3,414 loci was constructed. Compared to our previously published map of 2,247 loci [16], this map increased by 1,167 loci, with 541 new loci on the At subgenome and 626 new loci on the Dt subgenome. Previously, four genetic maps composed of more than two thousand loci in cotton have been reported [15-18]. Compared to these four earlier high-density maps, our newly updated map has the most loci (3,414 loci), the shortest distance between adjacent marker loci (average distance between loci is 1.08 cM), and the fewest number of gaps (a total of 16). In addition, ours is a gene-rich linkage map with 1,726 functional marker loci; 19 candidate genes islands, nine from the At subgenome and 10 from Dt subgenome, were also discovered. Considering the total map length, the updated map (3,667.62 cM) is shorter than two previously published maps from Rong et al. (2004) (4,447.9 cM) [15] and Yu et al. (2012) (4,418.9 cM) [18], and it is slightly longer compared that the map of Yu et al. (2011) (3,380 cM) [17].

The development of new markers was very important for construction of the new high-density genetic map. Retrotransposon-microsatellite amplified polymorphism (REMAP) markers have been described in some plants [57,58]; however, few reports have been published for cotton. Retrotransposons are very prevalent in the cotton genome [16]. Due to the accumulation of LTR retrotransposons, *Gossypium* genome size has undergone a threefold increase over the 5–10 Mya since its origin [12]. Thus, developing new markers related to retrotransposons will be important to define some regions of reduced recombination (cold-spot regions) of cotton chromosomes. Here, 188 polymorphic loci from 187 REMAP markers were anchored on the new genetic map, and a retrotranposon-rich region was found to be clustered with eight REMAP loci on D3 (Chr. 17). In the future, REMAP markers could be largely used to further enhance the saturation of cotton reference genetic maps in chromosomal heterochromatic regions.

### Segregation distortion regions are related to cotton evolution

Segregation distortion is increasingly being recognized as a potentially powerful evolutionary force [59] that may result from competition among gametes or from abortion of the gamete or zygote [60,61]. Of the 243 loci on the new map that showed distorted segregation, 152 (62.6%) were on the At subgenome, and only 93 loci (37.4%) on the Dt subgenome, even though more loci were tagged on the Dt subgenome (1,718 loci) than on the At subgenome (1,429 loci) in the newly constructed high-density genetic map. Thus, we speculate that the higher rate of polymorphism and the lower ratios of segregation in the Dt subgenome of tetraploid cotton may be a result of nucleocytoplasmic interactions [62]. Although more distorted loci were skewed toward the heterozygous allelic state than the homozygous state (129 vs. 114), the number of SDRs showing skewed transmission of *G. hirsutum* alleles exceeded the heterozygotes two-fold (8 vs. 4). One possible explanation was that *G. hirsutum* was the recurrent parent in our mapping population, and the pattern of transmission generally favored the elimination of the donor genotype, thus preserving the integrity of the recurrent genotype [62].

### Integrating genetic and cytogenetic maps will accelerate elucidation of chromosome structure in cotton

From the genetic map, we observed that DNA marker loci are distributed unevenly on the cotton chromosomes. Heterochromatic regions in chromosomes are well known to inhibit crossover formation [63]. The clustering of a great number of markers corresponding to the centromeric regions was recognized and physically verified in maize [64] and rice [65]. In cotton, the centromeres of Chr. 12 and Chr. 26 have been located on the cytogenetic map [37]. The marker loci BNL3816, NAU1237, and NAU2096 from Chr. 12 and BNL3816, NAU3006, NAU2356, and BNL840 from Chr. 26 are near the centromeric regions of these two chromosomes, respectively. In the newly updated genetic map, these markers that are linked to the centromeric region are distributed in the main peaks, indicating that the main peaks encompass the centromeric regions of the two chromosomes. Furthermore, in the newly updated genetic map, linkage groups A9, D5, and D9 had two main peaks, implying that there are two crossover suppression regions in each of these three chromosomes. Wang *et al.* (2008) distinguished the individual A-genome chromosomes by the BAC-FISH, and 45S rDNA and 5S rDNA probes gave hybridization signals on linkage groups A5, A7 and A9 [66]. The relationships between the rDNA regions and the two main peaks on these chromosomes needs to be further examined.

### Toward assembling the allotetraploid cotton genome

Tetraploid cotton (n = 2x = 26, AD) was derived from two diploids with A and D genomes that diverged from a common ancestor. The genus *Gossypium* consists of at least 45 diploid and five allotetraploid species [67]. The evolution of cotton species has been significantly affected by polyploidization events [7]. During evolution, all diploid cotton species originated from a common ancestor 5–15 million years ago, and all tetraploid cotton species originated 1–2 million years ago. As a consequence of polyploidization, when genes are duplicated they may continue to evolve at the same rate as they did in their diploid ancestors, or they may be subject to selection pressures that lead to differential rates of sequence change [68]. Ultimately, these duplicated sequences and their functions are maintained intact or undergo long-term evolutionary changes *via* sequence elimination [69,70], sequence rearrangement [71], gene silencing [72], or acquisition of new function [73]. Therefore, many paralogous loci, usually two homoeologous paralogous loci, one from the A-subgenome and another from the D-subgenome, occur as a result of polyploidization, with other paralogous loci arising from tandem duplications. Significant polyploidization may complicate the assembly of cotton genome sequences, especially if they are accompanied by frequent illegitimate recombination events that render 'islands' of paralogous DNA sequence (such as genes) homogeneous [74,75].

The high-density map described herein, and integration of cotton genomic data with genetically-mapped markers provides an excellent bridge to assemble cotton genome sequences accurately, fine map tagged QTL, and accomplish the confirmation of genes structure and function [76,77]. In this study, loci duplication, rearrangement and translocation were all detected by the analysis of duplicated loci. Nevertheless, using a PCR-based computational method, a large number of cotton EST and BAC sequences were anchored to the cotton genetic map based on the available marker primer probes. In the bioinformatics analysis, we found that a tolerance of three mismatches in the alignments achieved a good balance between performance and accuracy. Therefore, locus applicability could be greatly enhanced by identifying the corresponding gene functions. As a result, 337 ESTs/genes related to fiber quality traits were integrated with 132 previously reported cotton fiber quality QTL, with more on the D-subgenome than on the A-subgenome. This finding indicated that the D-subgenome from a non-fiber-production ancestor plays a large role in the genetic control of fiber growth and development in polyploid cotton. The ESTs/genes from the D-subgenome were important for improving cotton fiber quality, and these could be studied in depth to elucidate the relationship between ESTs/genes and QTL related to important fiber traits, further for the improvement of fiber quality in breeding purposes. The importance of the D-subgenome in lint fiber development has also been previously studied by a meta-analysis of polyploid cotton fiber QTL [78] and a joint analysis of multiple backcross generations [79].

The 2,111 previously-mapped independent markers were successfully matched to EST sequences and BAC clones from different cotton species or the two subgenomes in tetraploid cotton species. Furthermore, we detected higher-level evolutionary sequence conservation in the different *Gossypium* species, as well as sequence size differences of paralogous and orthologous loci in the natural evolutionary process of genus *Gossypium*. The integrated physical sequences and the genetic map provide us with valuable resources for comparative genomics of different cotton species, for distinguishing the two different subgenomes from one another, and for ultimately elucidating the genomic determinants of phenotypic diversity between cotton species that evolved within the last 5–15 Mya.

## Conclusion

In conclusion, the construction of a high-density linkage map provides an essential resource to facilitate the correct ordering of DNA segments on chromosomes for the comprehensive and accurate assembly of the allotetraploid cotton genome, and will enable further clarification of genome organization changes revealed by multiple species comparisons. The future availability of whole-genome sequences from cotton species will provide us with an unprecedented opportunity to analyze features of genome organization at the DNA sequence level, to study differences between organisms by comparing whole genomic sequences, and to enhance our understanding of the functional and agronomic significance of polyploidy and genome size variation in *Gossypium*.

## Methods

### Sources of primers

To refine our previously-constructed genetic linkage map of tetraploid cotton, we screened more than 2000 primer pairs. In detail, 1,000 new SSR primer pairs with prefixes GH from Texas A&M University, CER, CGR, COT, DC, DPL, and SHIN from Monsanto and HAU from Huazhong Agricultural University (http://www.cottonmarker.org/) were chosen. In addition, 726 new eSSR primers pairs, designated ‘NAU’ for Nanjing Agricultural University, were developed using non-redundant EST sequences from *G. barbadense* cv. Hai7124 and *G. raimondii*; The other primers, including RT (PCR amplification of cDNA sequences), CAPs (cleaved amplified polymorphisms), BAC-end (BAC end sequences) and SNP (single nucleotide polymorphisms) are designed ‘Y’ or have the gene name itself as the primer prefix; these were developed based on known gene or BAC end sequences. Polymorphic InDel loci, where InDel primers were developed based on known EST sequences, were directly tagged on the linkage maps with the prefix ‘InDel’. We also developed retrotransposon-microsatellite amplified polymorphism (REMAP) markers in cotton by randomly combining long terminal repeat (LTR)-specific primers with simple sequence repeat (SSR) primers.

### Plant material, DNA extraction, PCR amplification, and electrophoresis

The mapping population was composed of 138 BC_1_ individuals that were generated from the cross [(TM-1 × Hai7124) × TM-1] [34]. TM-1 is a genetic standard line of Upland cotton and Hai7124 is a commercial Sea island *Verticillium*-resistant cultivar. Cotton genomic DNA was isolated from the two parents and each BC_1_ individual as described by Paterson et al. [80]. SSR-PCR amplifications were performed using a Peltier Thermal Cycler-225 (MJ Research) and electrophoresis of the products was performed as described by Zhang et al. [81,82].

### Construction of the genetic linkage map

All primer pairs were first used to screen the parental lines TM-1 and Hai7124 for polymorphisms. Polymorphic markers were then used to survey 138 individuals of the BC_1_ mapping population. The maternal (TM-1) genotype and the heterozygous (F_1_) genotype were scored as 1 and 3 in the BC_1_ population, respectively. Missing data were noted as “-”. The χ^2^ test for goodness of fit was used to assess the Mendelian 1:1 inheritance expected in the BC_1_ segregating population.

JoinMap 3.0 [83] was used to calculate the genetic linkage map. The Kosambi mapping function [84] was used to convert recombination frequencies to genetic map distances (centimorgan, cM). All linkage groups were determined at log-of-odds (LOD) scores ≥6. Linkage groups were assigned to chromosomes on the basis of our backbone linkage maps [16,31-33] and the results of BAC–FISH [fluorescence in situ hybridization (FISH) using bacterial artificial chromosome (BAC) clones as probes] [85]. Chromosome nomenclature was referenced to our previously published chromosome naming system [85].

### *Gossypium* ESTs and genomic sequence resources

In the present study, a total of 413,113 available ESTs and 195 BACs in the NCBI GenBank database (http://www.ncbi.nlm.nih.gov) were extracted and organized. The ESTs were mainly from four major cotton species (*Gossypium hirsutum*, *G. barbadense*, *G. raimondii*, and *G. arboreum*). All physical sequences were trimmed to remove vector, adapter and low complexity regions based on the UniVec (http://www.ncbi.nlm.nih.gov/Univec) and RepBase databases [86] using stringent cutoff parameters. Approximately 410,102 cleaned ESTs and 195 BACs were used for the further integration analysis.

### Integration of genetic markers and genomic DNA sequence information

To align the markers with the EST and BAC sequences onto the genetic map, 3,244 informative sequence-based molecular markers were used. A PCR-based *in silico* screening procedure was carried out with stringent cutoff parameters: mismatch ≤3, and FR (Forward-Reverse primer pair sequences) match pattern to ensure the specificity using Perl script program. This query sequence was also searched against the BAC sequence database using a hashing algorithm to identify high-scoring segment matches with a paired-end match pattern. High scoring hits were then extended in each direction until the sequence similarity score fell below a threshold or one of the separation characters was encountered.

### Putative gene ontology and metabolic pathway analysis

The represented unigenes were subjected to a homology analysis against the NCBI RefSeq plant protein database (http://www.ncbi.nlm.nih.gov/RefSeq/, release 53, May 10, 2012) using the Blastx alignment program [87]. Blastx searches were performed at an E value of 1e-05 to filter out nonspecific high-scoring segment pairs. Different descriptive statistics charts for the results of the Blast alignments were then assigned for alignment evaluation.

The set of unigenes was submitted for GO (gene ontology) annotation using the Blast2GO program with the default parameters [88,89]. The program extracted the GO terms associated with homologies identified with BLAST and returned a list of GO annotations represented as hierarchical categories of increasing specificity.

Unigenes were assigned to metabolic pathways with the tools supplied by the Kyoto Encyclopedia of Genes and Genomes (KEGG) [90]. The unigenes were processed using the bi-directional “best hit” method (forward and reverse reads) to assign orthologs. KAAS (KEGG Automatic Annotation Server, http://www.genome.jp/kegg/kaas/) provided a functional annotation of putative genes by Blast comparisons against the KEGG GENES database. The output included KO (KEGG Orthology) assignments and automatically generated KEGG pathways.

### Integrating ESTs/genes with previously reported QTL related to fiber quality

Fiber quality QTL previously reported in our lab [39-53] were chosen for the analysis of these QTL chromosome distribution characteristics and to reveal the relationship between these QTL and ESTs/genes related to fiber development. Integration was performed according to the marker interval and QTL peak location information. Only the QTL region flanking markers within 20 cM were selected to mine the ESTs/genes in the region. The fiber quality traits mainly included fiber length (FL), fiber strength (FS), fiber fineness (FF), fiber elongation (FE) and fiber uniformity (FU). To cluster QTL from different populations, the meta-analysis was carried out using the “Meta-Analysis” function in the BioMercator v 2.1 software program [91].

## Abbreviations

BAC: Bacterial artificial chromosome; FISH: Fluorescence in situ hybridization; InDel: Insertion-deletion; QTL: Quantitative trait loci; REMAP: Retrotransposon-microsatellite amplified polymorphism; SSR: Simple sequence repeats; SNP: Single nucleotide polymorphism; FL: Fiber length; FS: Fiber strength; FF: Fiber fineness; FE: Fiber elongation; FU: Fiber uniformity.

## Competing interests

The authors have declared that no competing interests exist.

## Authors’ contributions

WZG designed the experiments. WZG, LZ and YDL conceived the experiments and analyzed the results. LZ carried out most of the experiments, YDL and CPC carried out all computational analyses. XCT, XDC, WZ, HD, and XHG participated in part of mapping experiments. LZ, YDL and WZG drafted the manuscript and WZG revised the manuscript. All authors read and approved the final manuscript.

## Supplementary Material

Additional file 1 **Table S1. **Chromosome information in the newly updated high-density genetic map. Click here for file

Additional file 2 **Table S2. **Details of the duplicated SSR loci and their chromosomal locations. Click here for file

Additional file 3 **Figure S1. **The frequency distribution of polymorphic loci in each chromosome. Click here for file

Additional file 4 **Table S3. **The cluster position, and numbers and types of loci in the cluster. Click here for file

Additional file 5 ** Table S4. **Gene numbers predicted in each chromosome by genetically and physically anchored EST and BAC resources. Click here for file

Additional file 6 **Figure S2. **Functional annotation of 2,748 represented unigenes. Click here for file

Additional file 7 **Figure S3. **Descriptive statistical analysis of the Blast alignment results against the NCBI RefSeq plant database. Click here for file

Additional file 8 **Table S5. **Functional annotation and classification summary for the 2,748 unigenes. Click here for file

Additional file 9 **Figure S4. **Functional classification of the 2,748 unigenes that were assigned GO terms. Click here for file

Additional file 10 **Table S6. **Functional classification of 2,748 unigenes based on KEGG. Click here for file

Additional file 11 ** Figure S5. **Functional classification of the 2,748 unigenes that were assigned level 2 KEGG metabolism terms. Click here for file

Additional file 12 **Table S7. **Information on ESTs/genes integrated on fiber-related QTLs clustered within a 40-cM interval on the D8 chromosome. Click here for file
