# Supplementary material for: Toward allotetraploid cotton genome assembly: integration of a high-density molecular genetic linkage map with DNA sequence information
Source: BMC Genomics. 2012 Oct 9;13:539. doi: 10.1186/1471-2164-13-539 (PMC3557173; doi:10.1186/1471-2164-13-539)
Supplement: Additional file 3 — Figure S1. The frequency distribution of polymorphic loci in each chromosome. [file 1471-2164-13-539-S3.pdf]

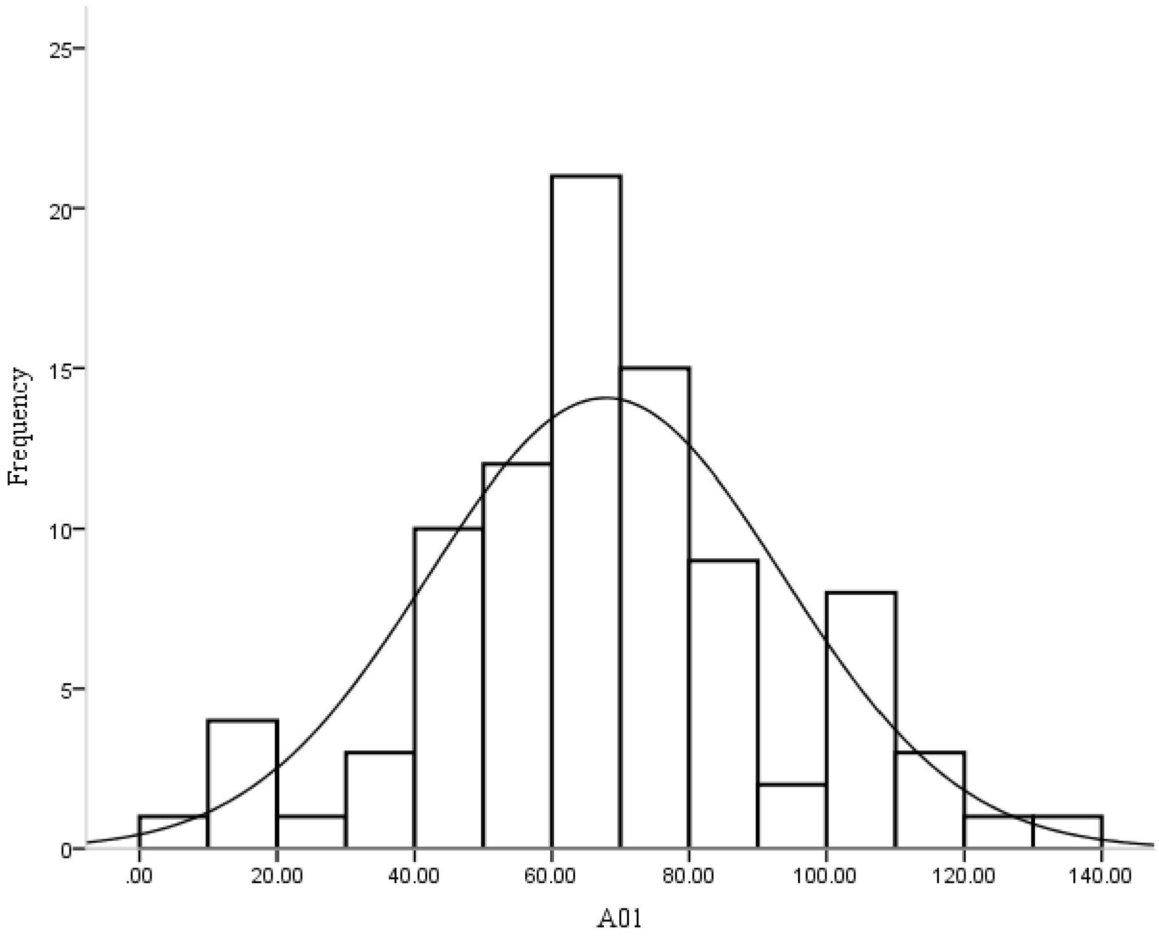

25
20
15
10
5
0
Frequency
.00
20.00
40.00
60.00
80.00
100.00
120.00
140.00
A01

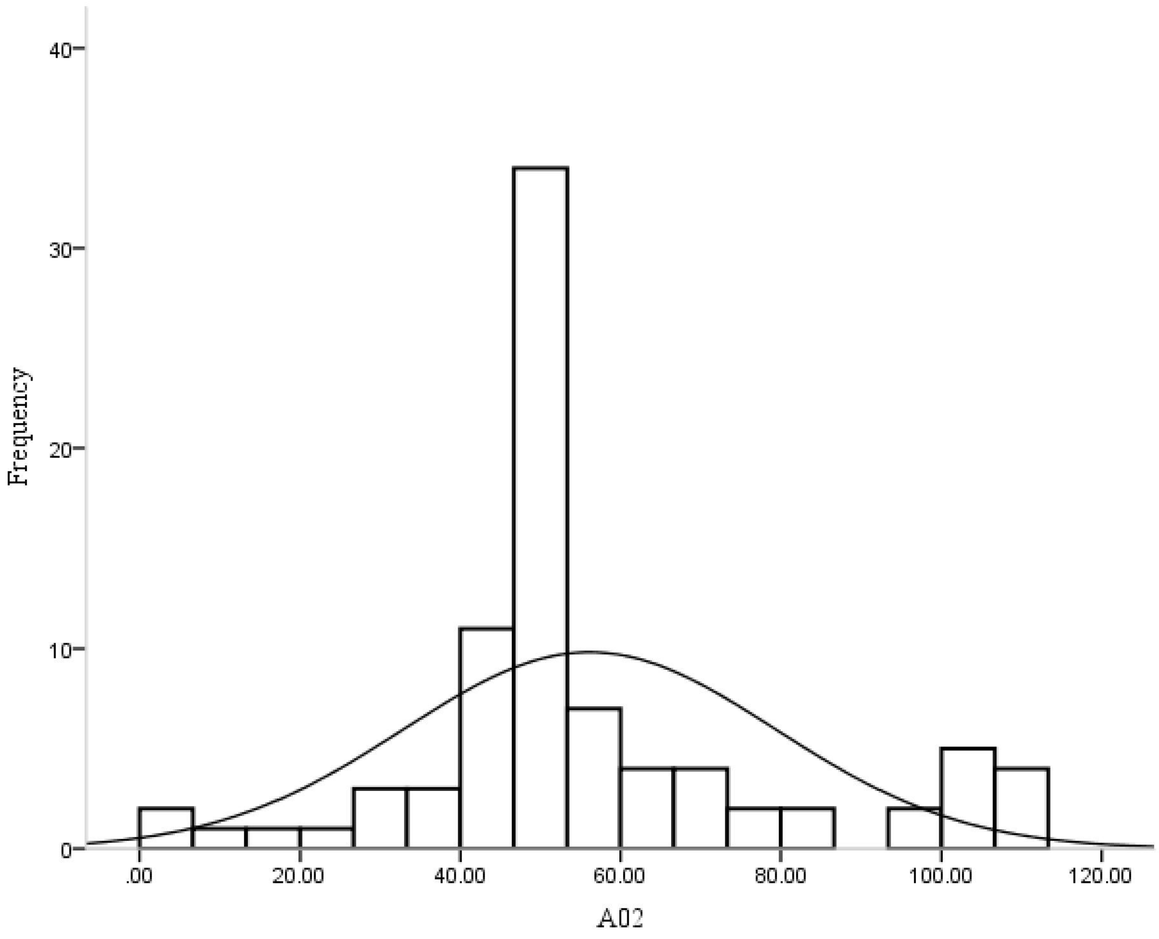

Frequency
40
30
20
10
0
.00
20.00
40.00
60.00
80.00
100.00
120.00
A02

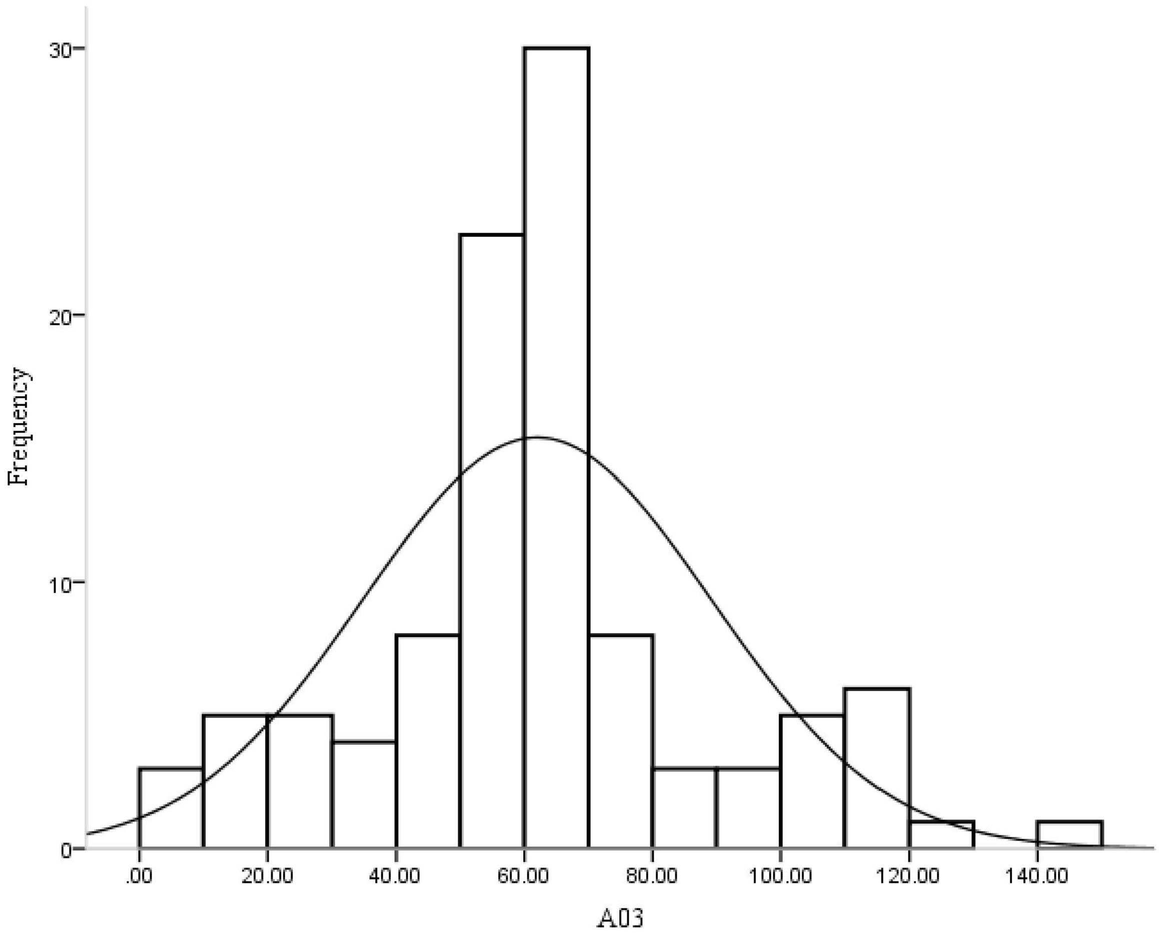

Frequency
30
20
10
0
.00
20.00
40.00
60.00
80.00
100.00
120.00
140.00
A03

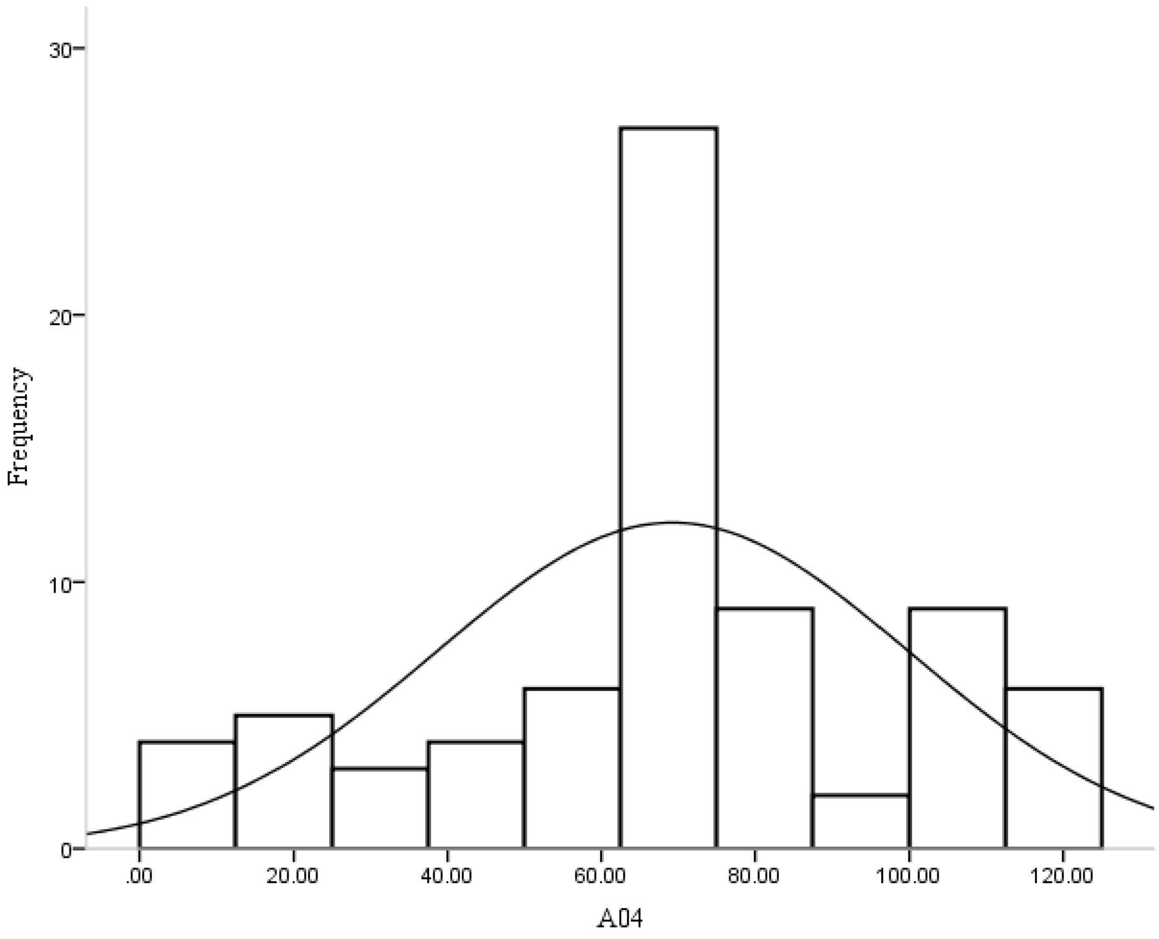
30
20
10
0
Frequency
.00
20.00
40.00
60.00
80.00
100.00
120.00
A04

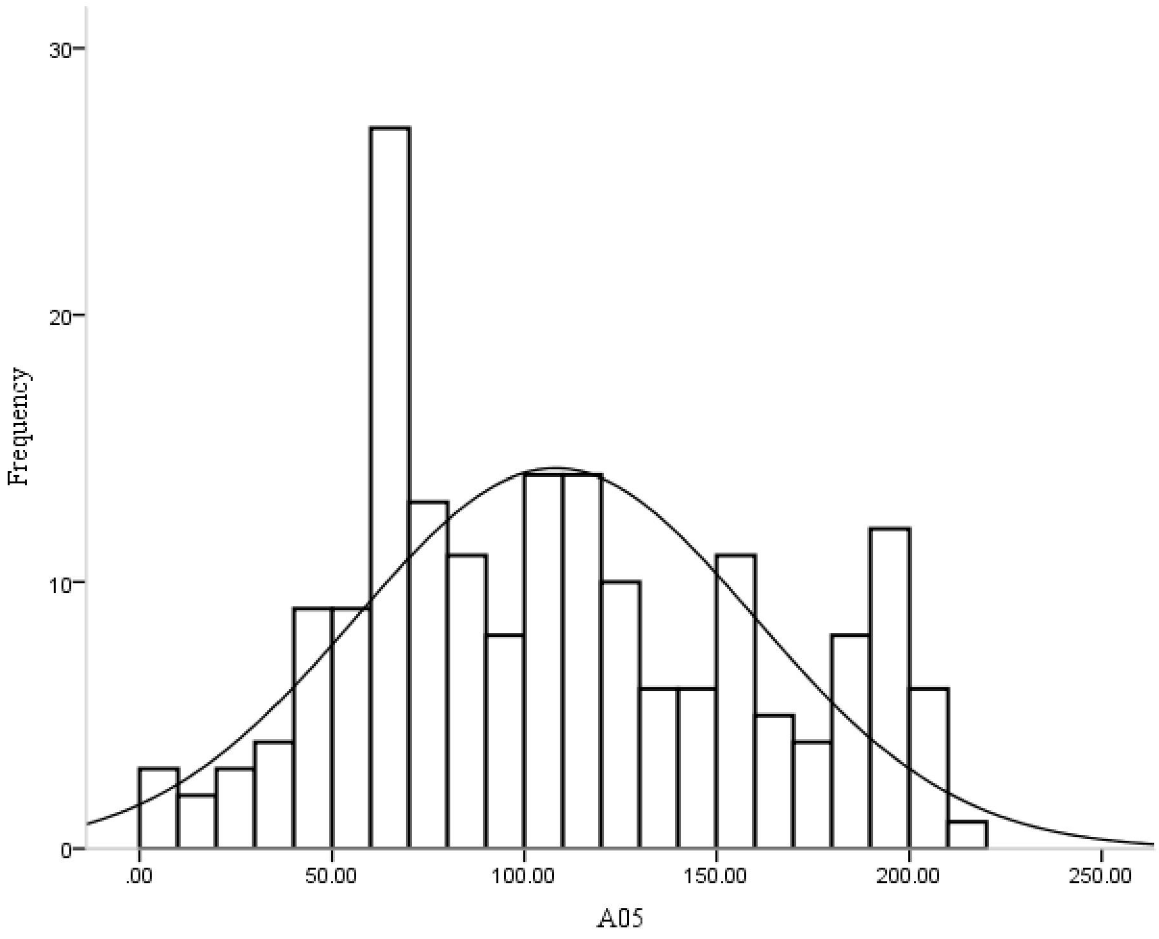

Frequency
30
20
10
0
.00
50.00
100.00
150.00
200.00
250.00
A05

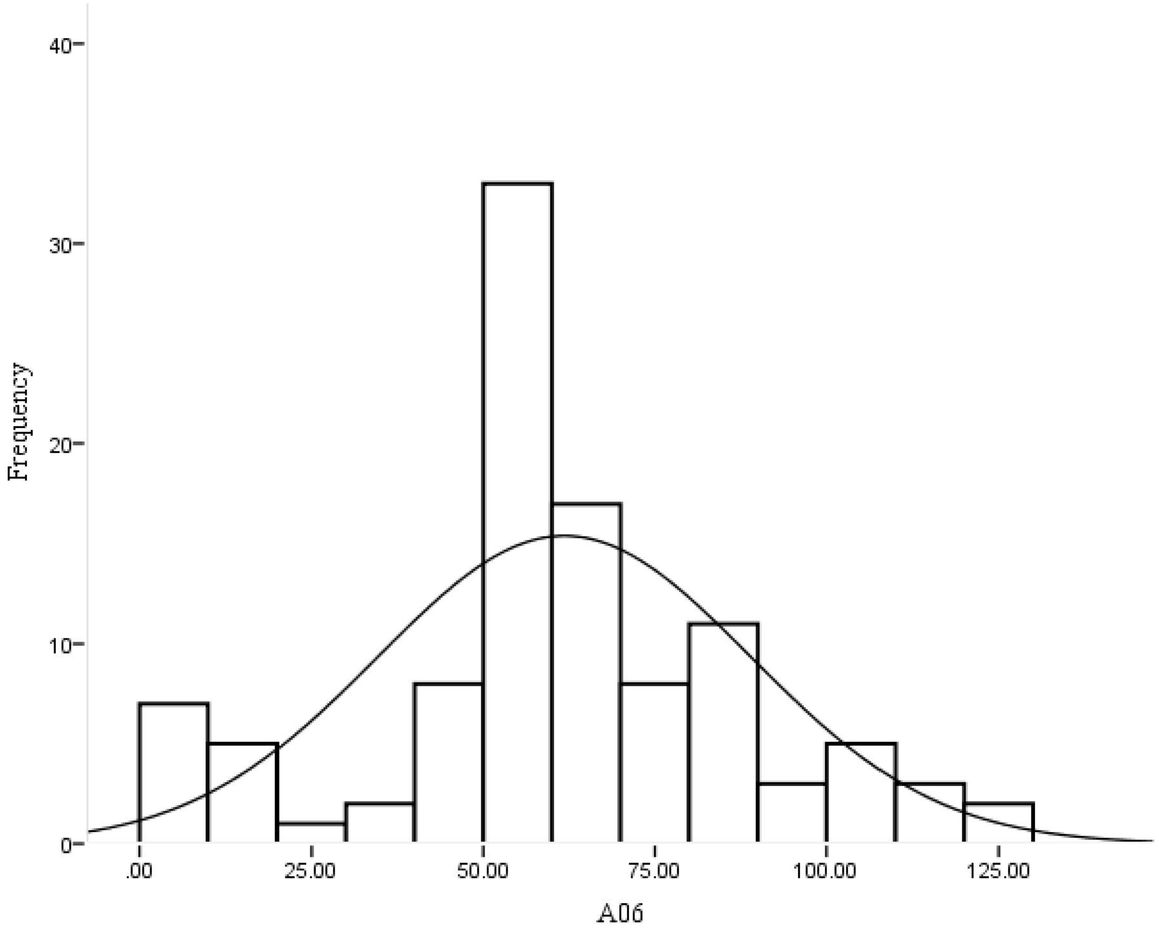
40
30
20
10
0
Frequency
.00
25.00
50.00
75.00
100.00
125.00
A06

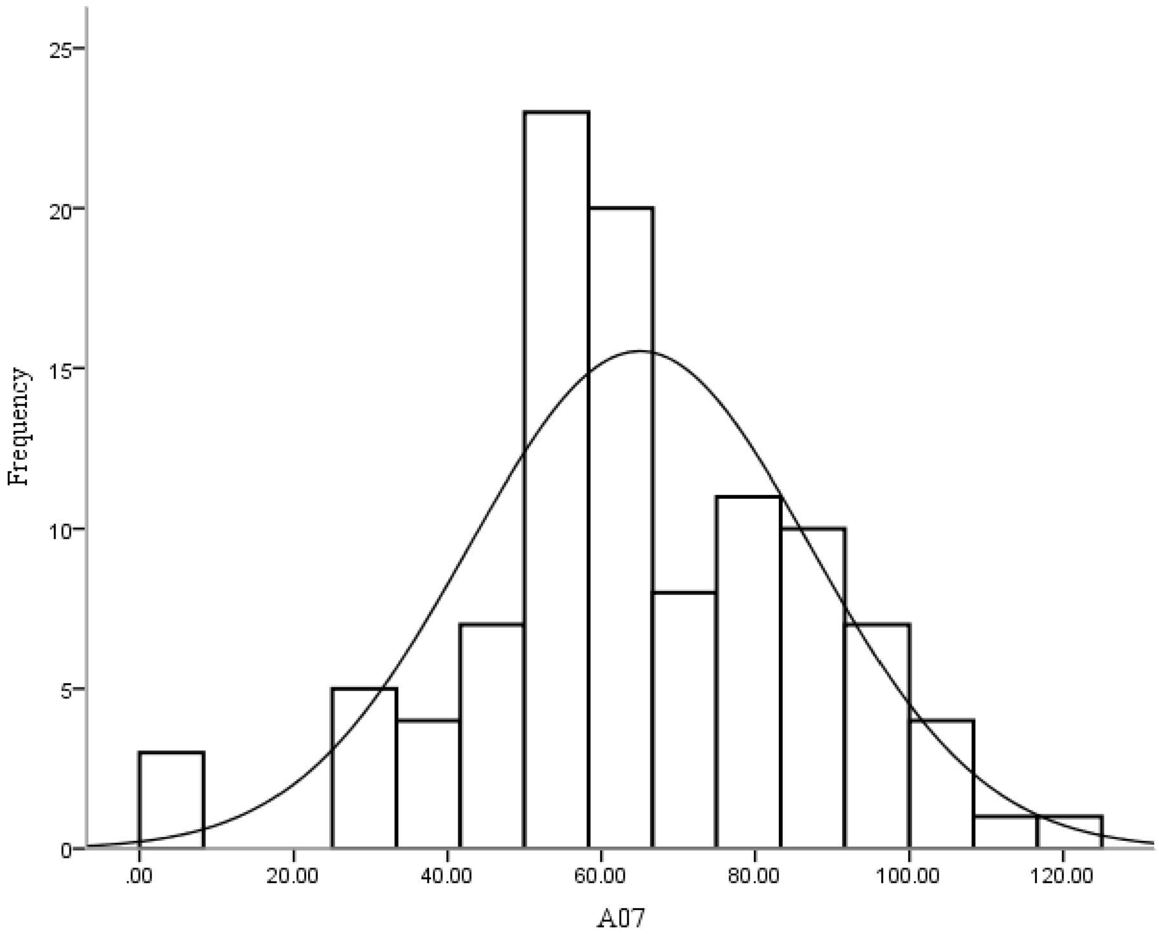

Frequency
25
20
15
10
5
0
.00
20.00
40.00
60.00
80.00
100.00
120.00
A07

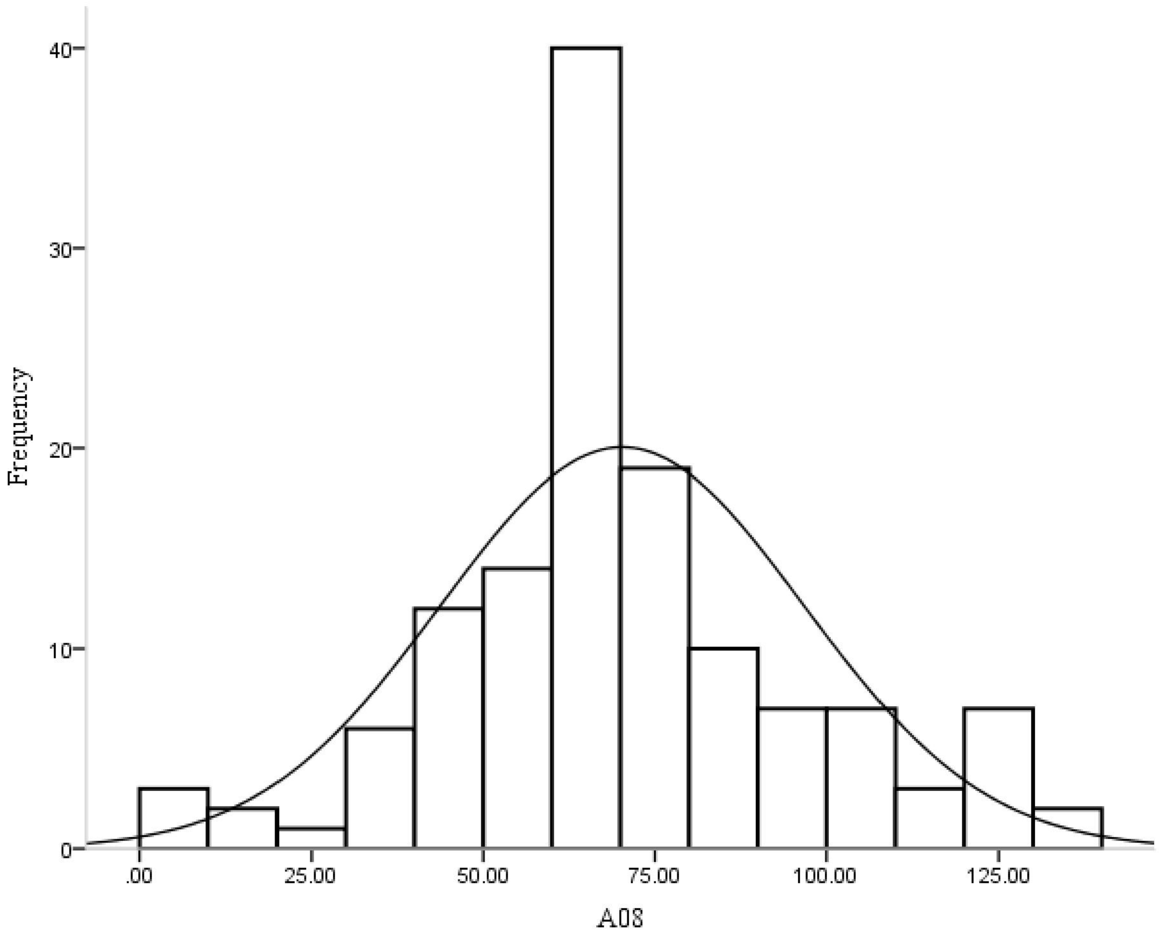

40
30
20
10
0
Frequency
.00
25.00
50.00
75.00
100.00
125.00
A08

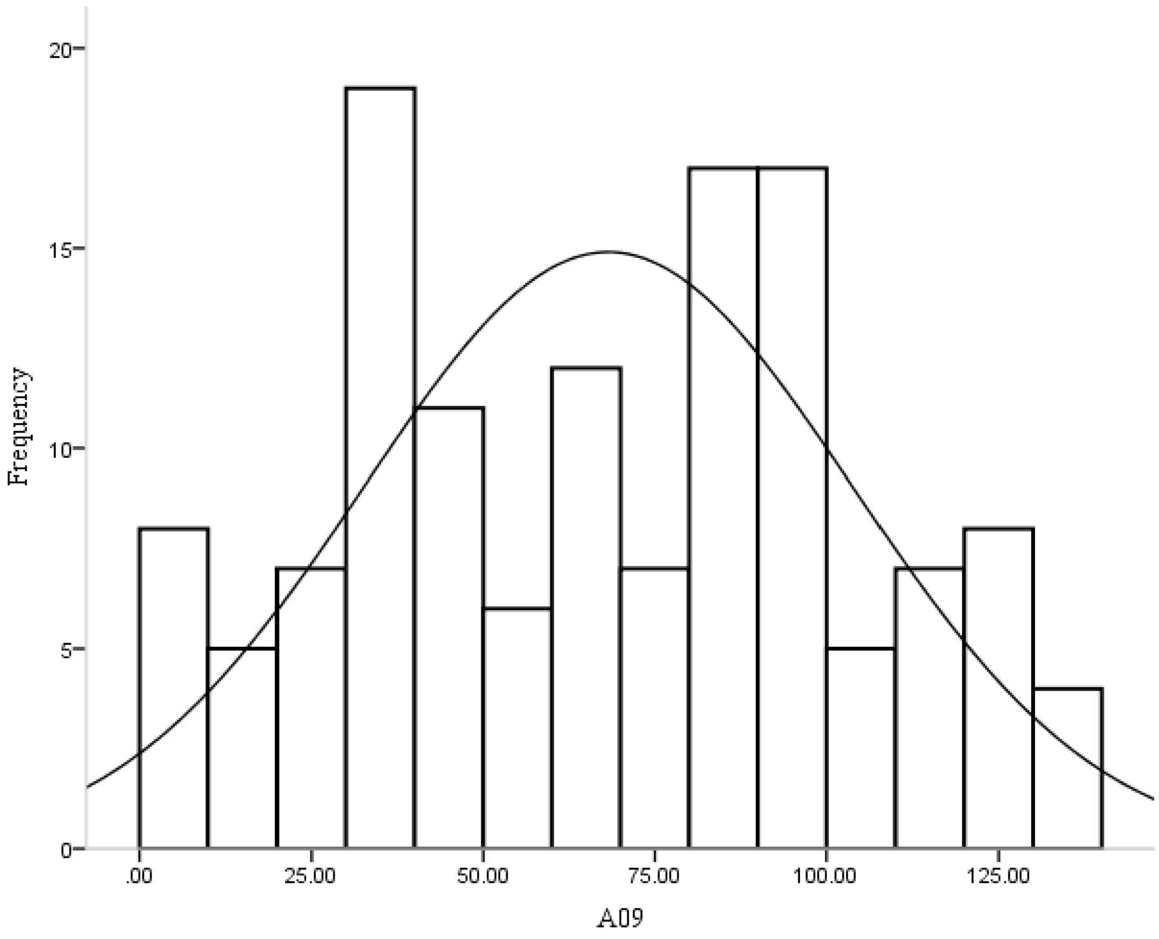

20
15
10
5
0
Frequency
.00
25.00
50.00
75.00
100.00
125.00
A09

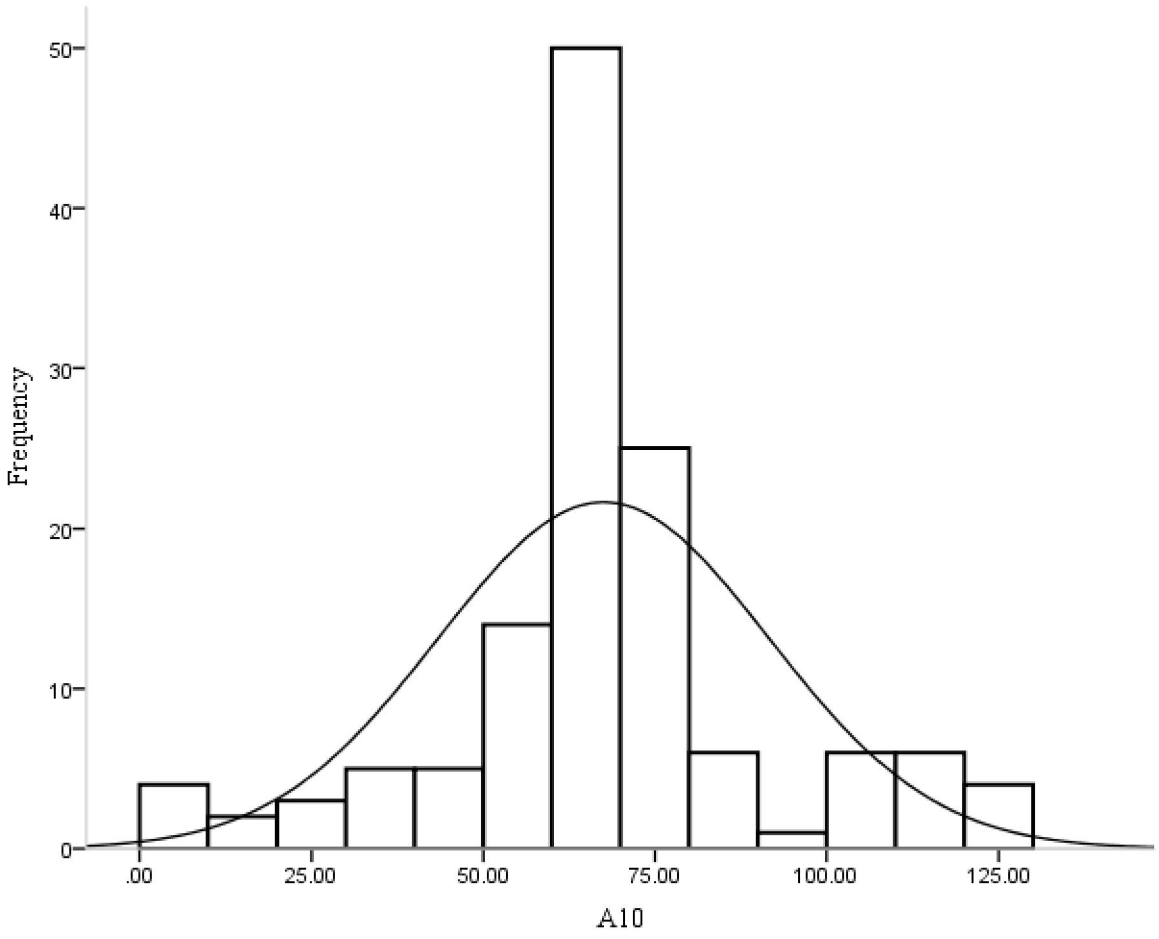

50
40
30
20
10
0
Frequency
.00
25.00
50.00
75.00
100.00
125.00
A10

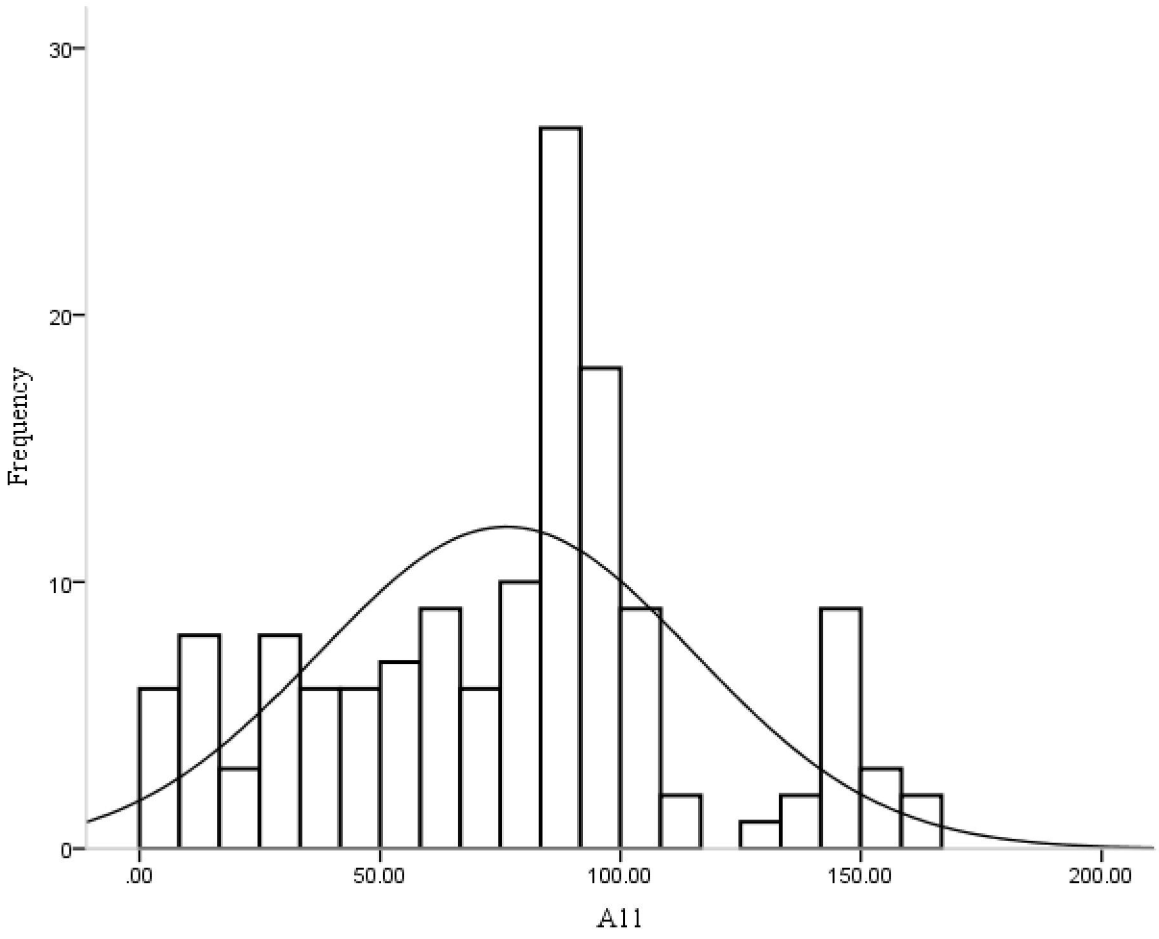

30
20
10
0
Frequency
.00
50.00
100.00
150.00
200.00
A11

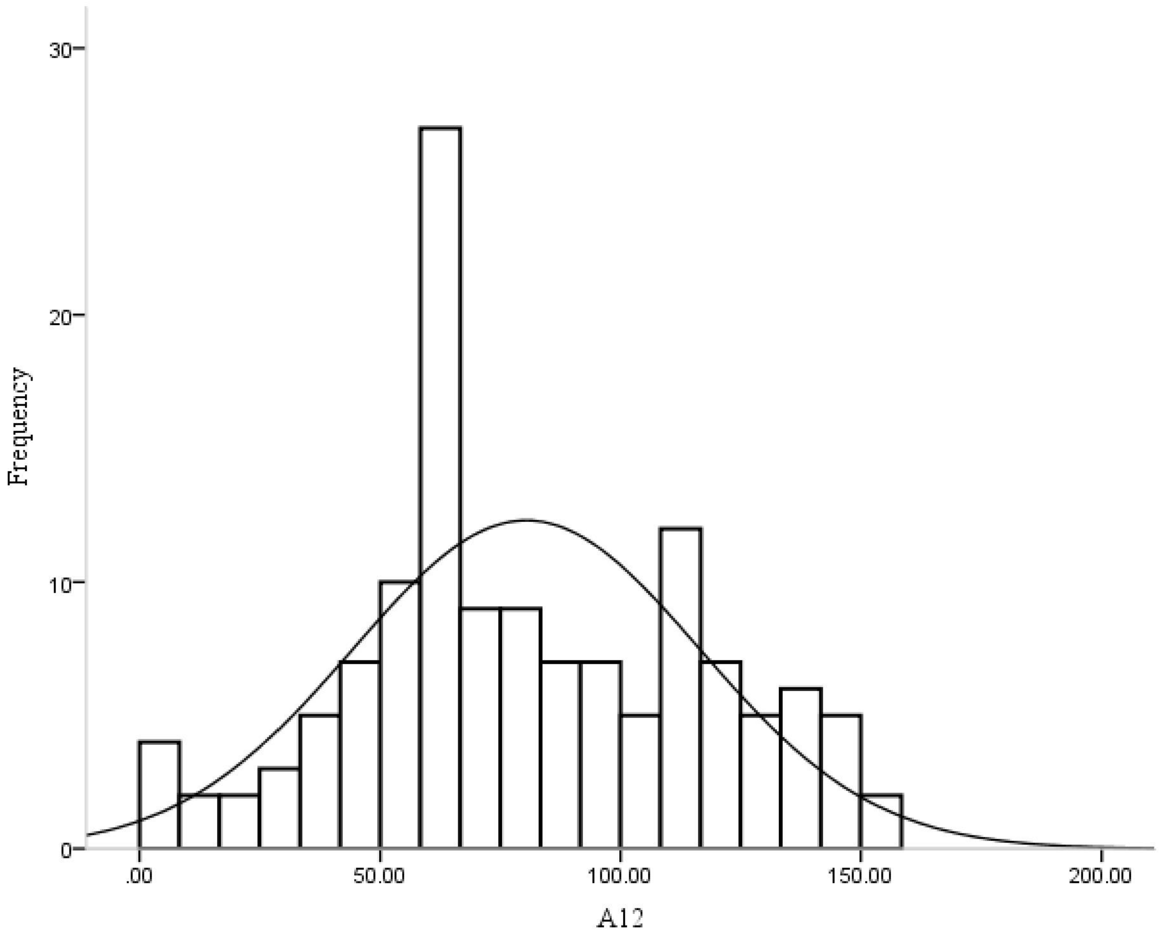

30
20
10
0
Frequency
.00
50.00
100.00
150.00
200.00
A12

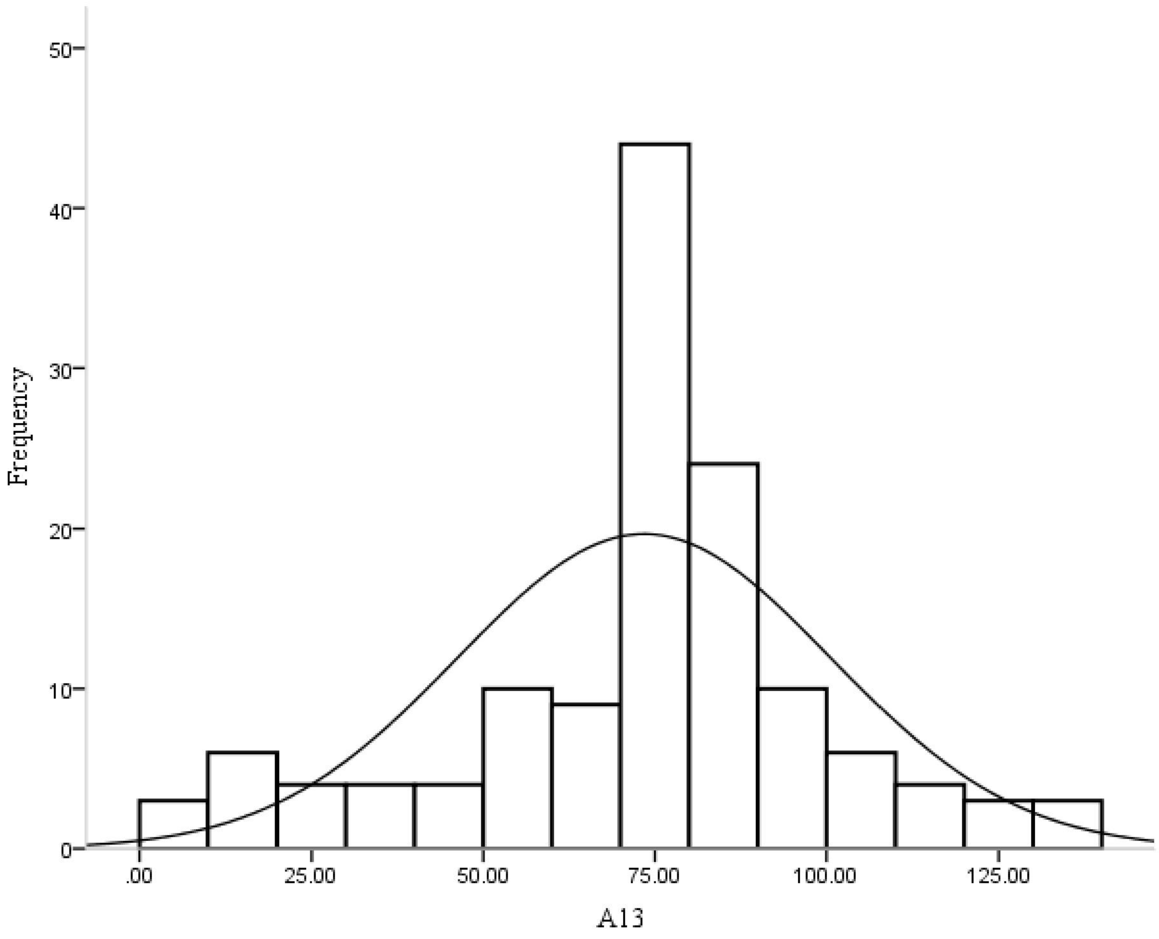

50
40
30
20
10
0
Frequency
.00
25.00
50.00
75.00
100.00
125.00
A13

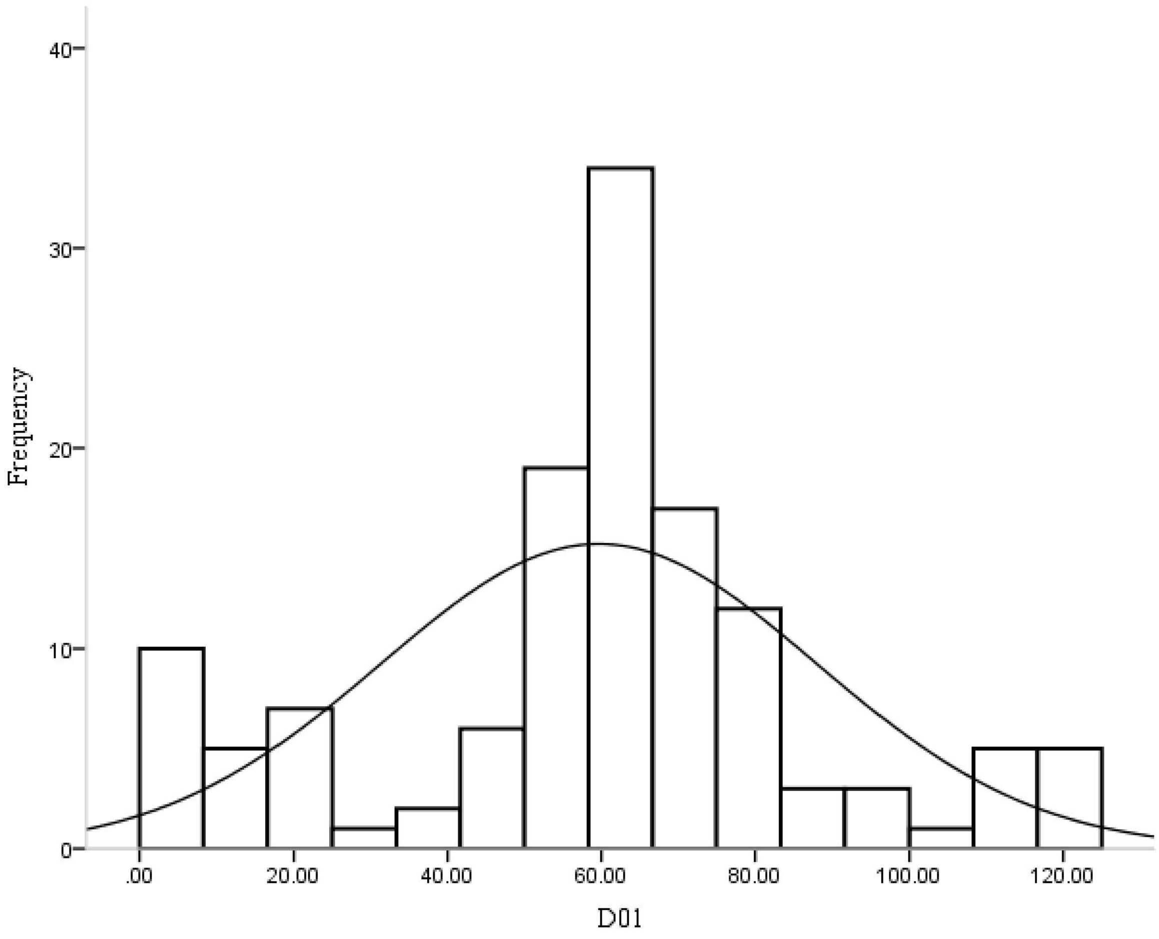
Frequency
40
30
20
10
0
.00
20.00
40.00
60.00
80.00
100.00
120.00
D01

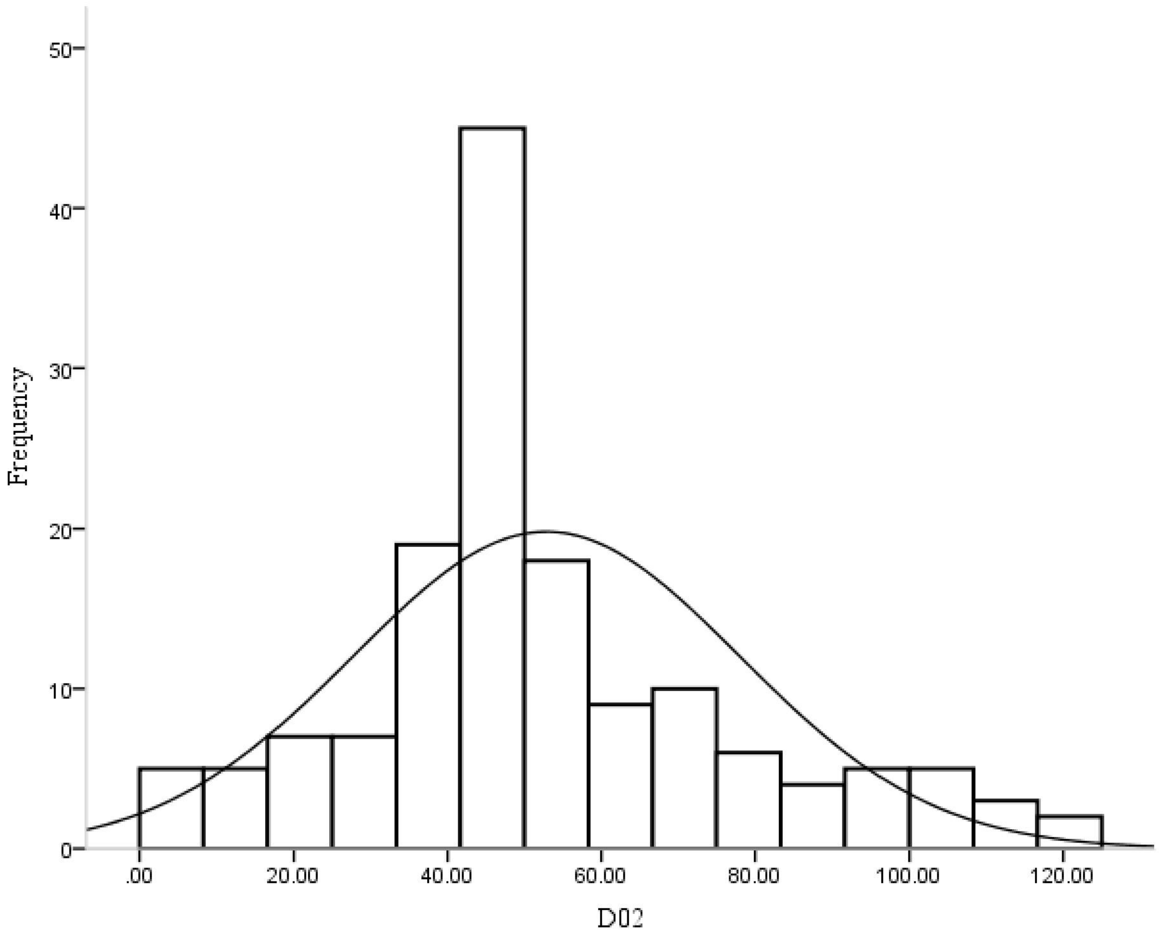

Frequency
50
40
30
20
10
0
.00
20.00
40.00
60.00
80.00
100.00
120.00
D02

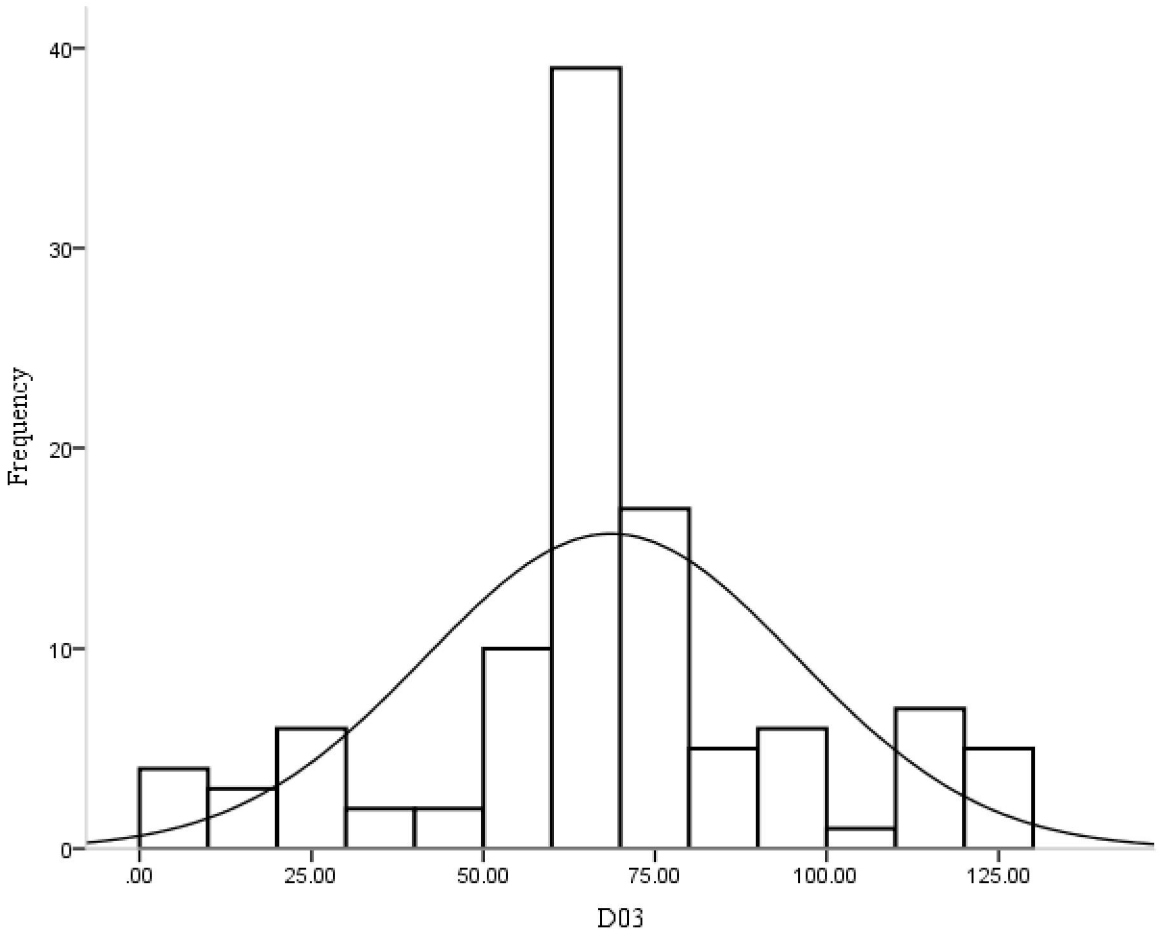

40
30
20
10
0
Frequency
.00
25.00
50.00
75.00
100.00
125.00
D03

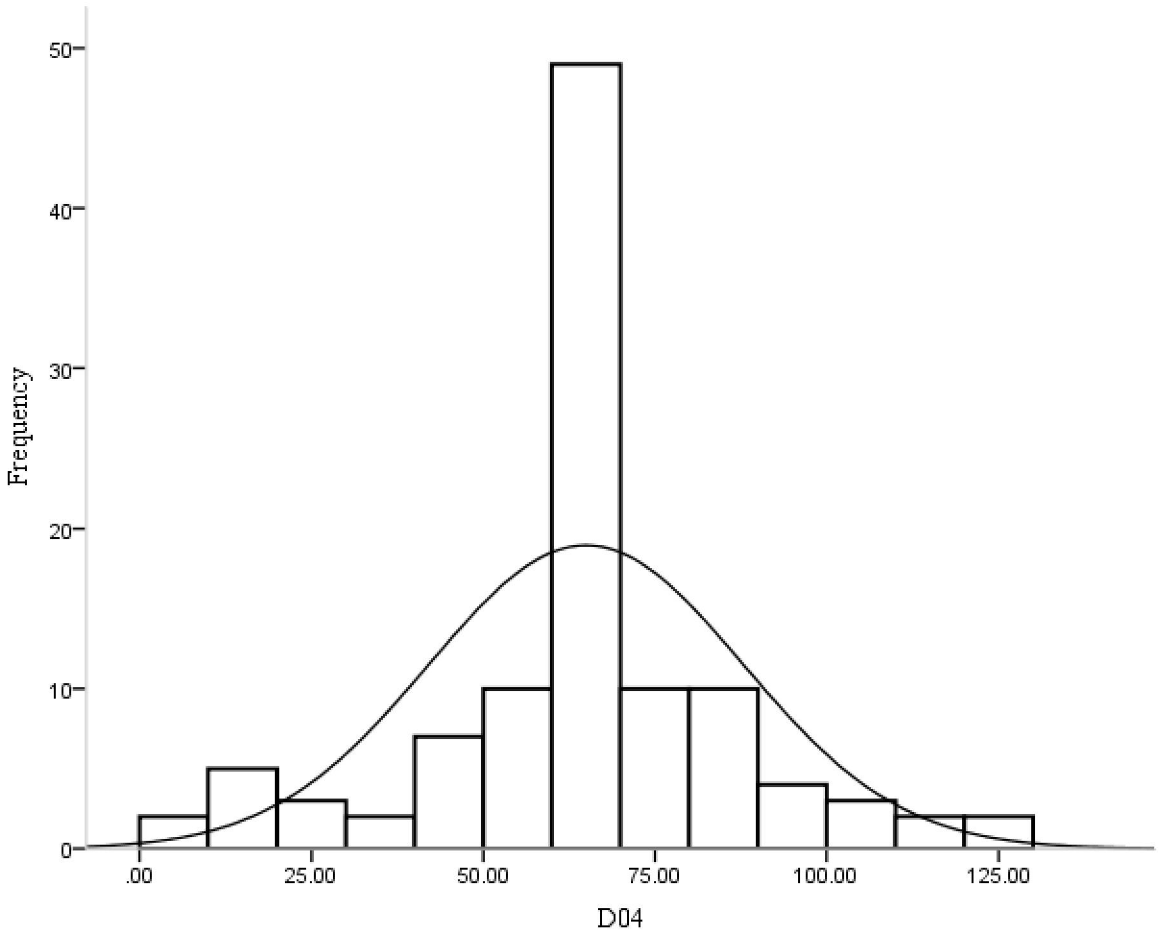

Frequency
50
40
30
20
10
0
.00
25.00
50.00
75.00
100.00
125.00
D04

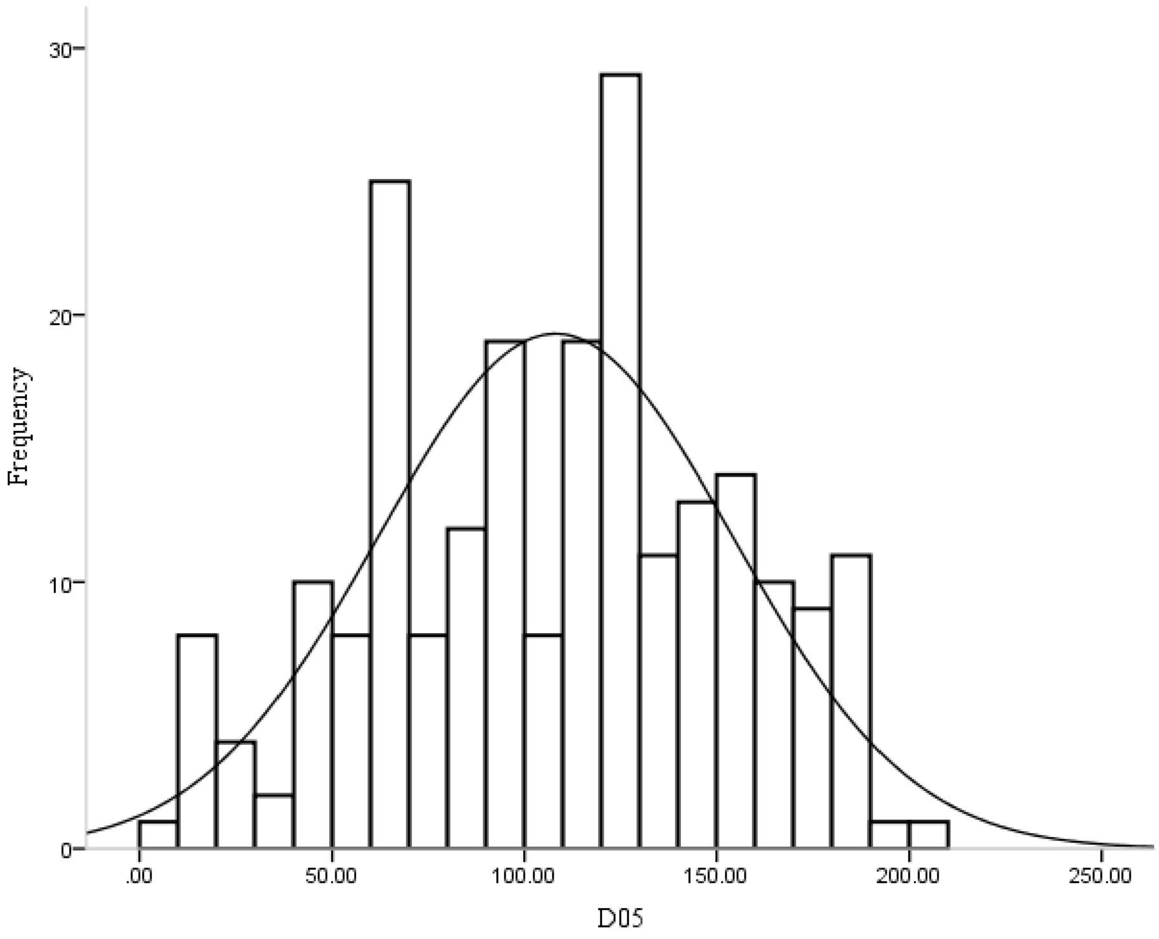

30
20
10
0
Frequency
.00
50.00
100.00
150.00
200.00
250.00
D05

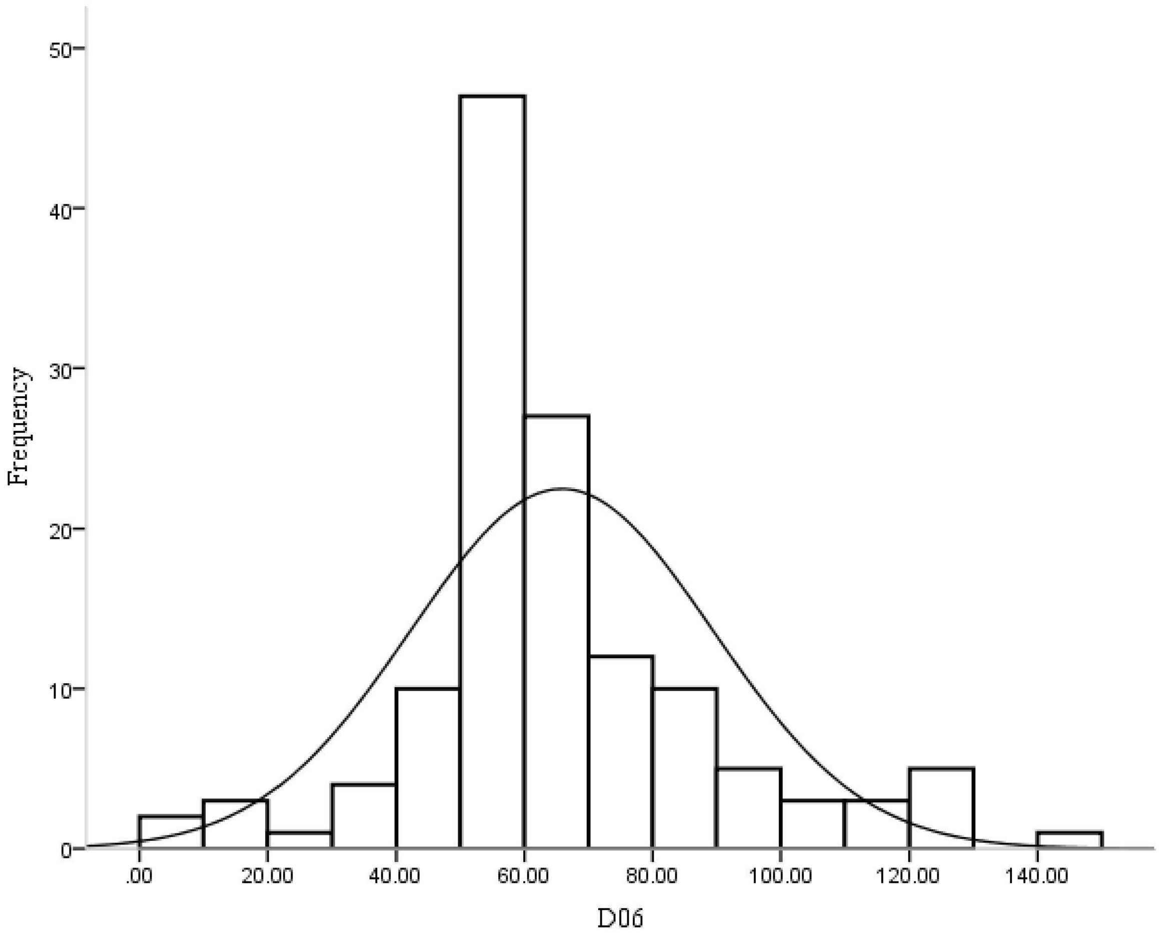

50
40
30
20
10
0
Frequency
.00
20.00
40.00
60.00
80.00
100.00
120.00
140.00
D06

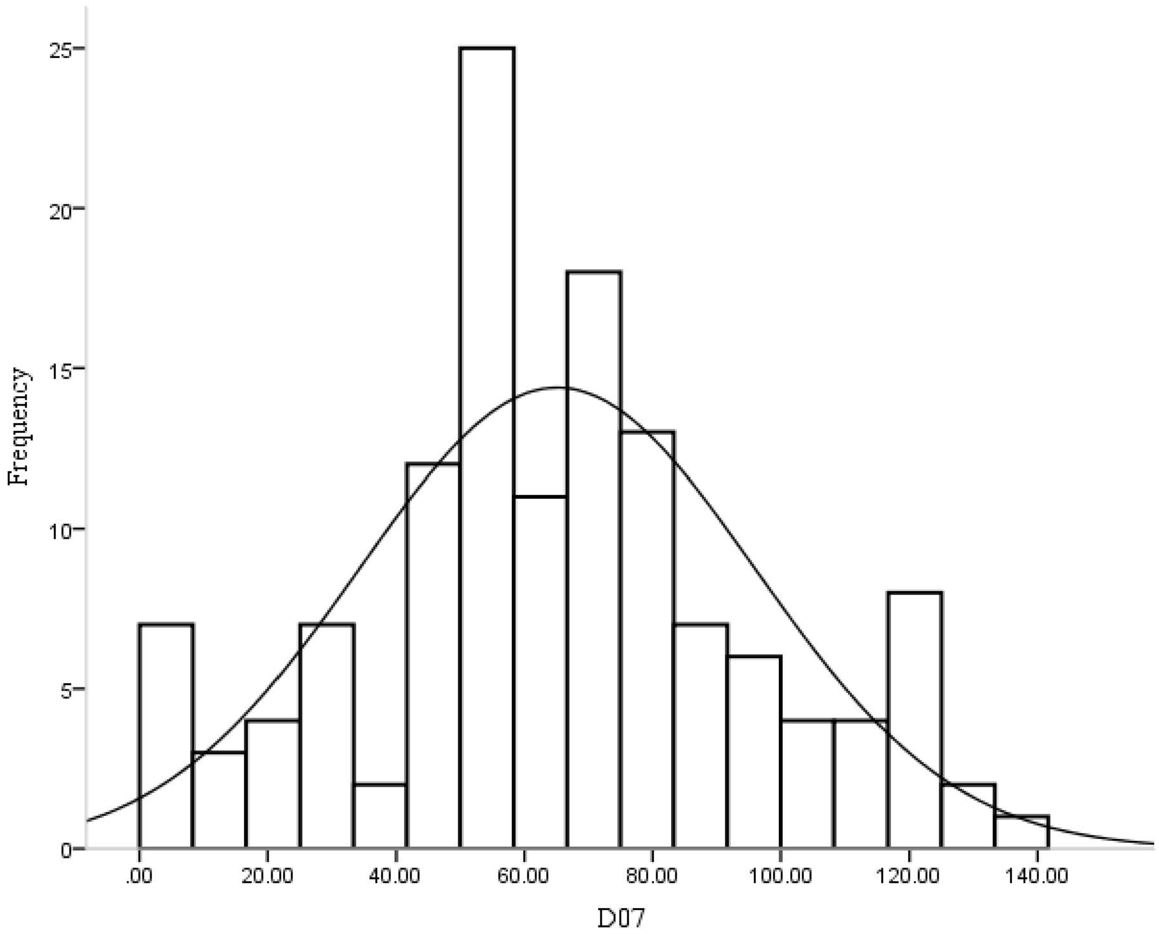

25
20
15
10
5
0
Frequency
.00
20.00
40.00
60.00
80.00
100.00
120.00
140.00
D07

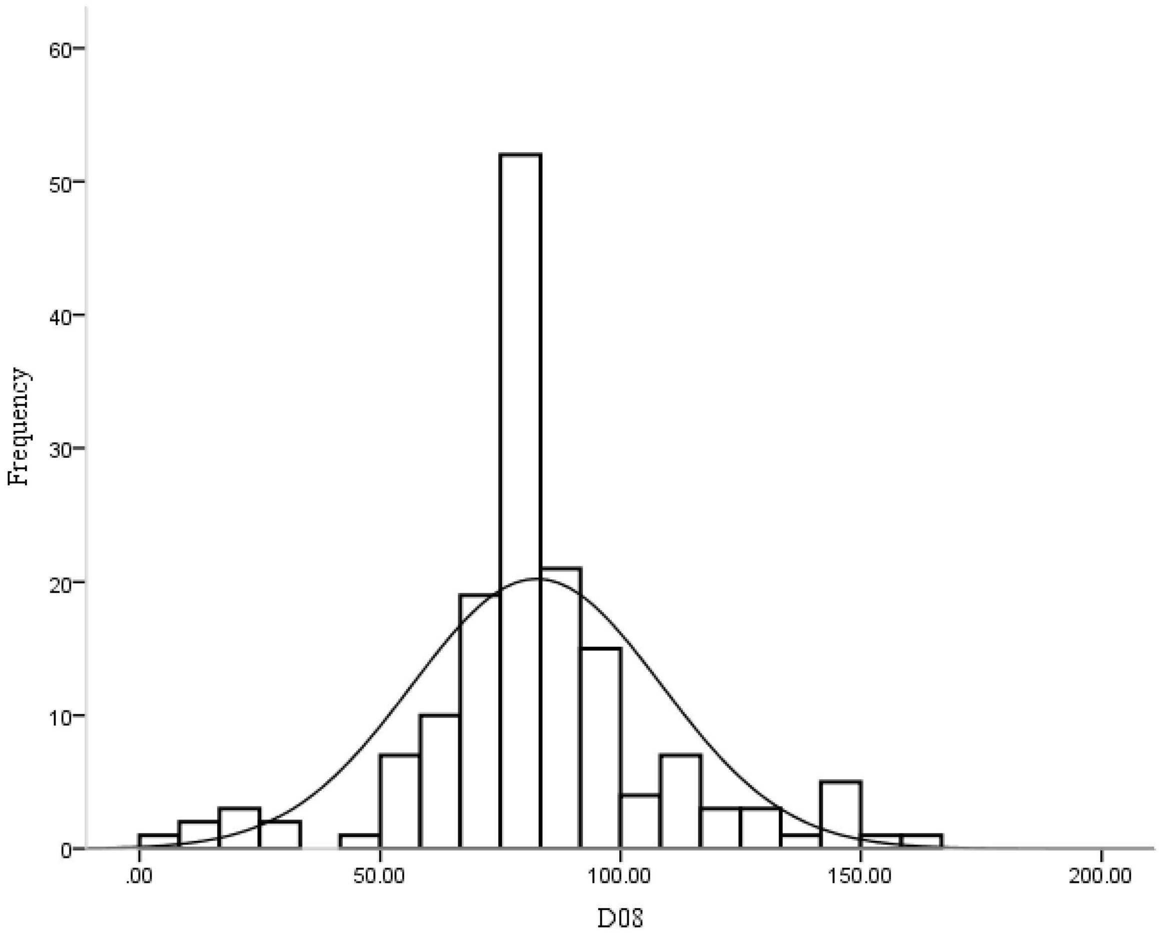

Frequency
60
50
40
30
20
10
0
.00
50.00
100.00
150.00
200.00
D08

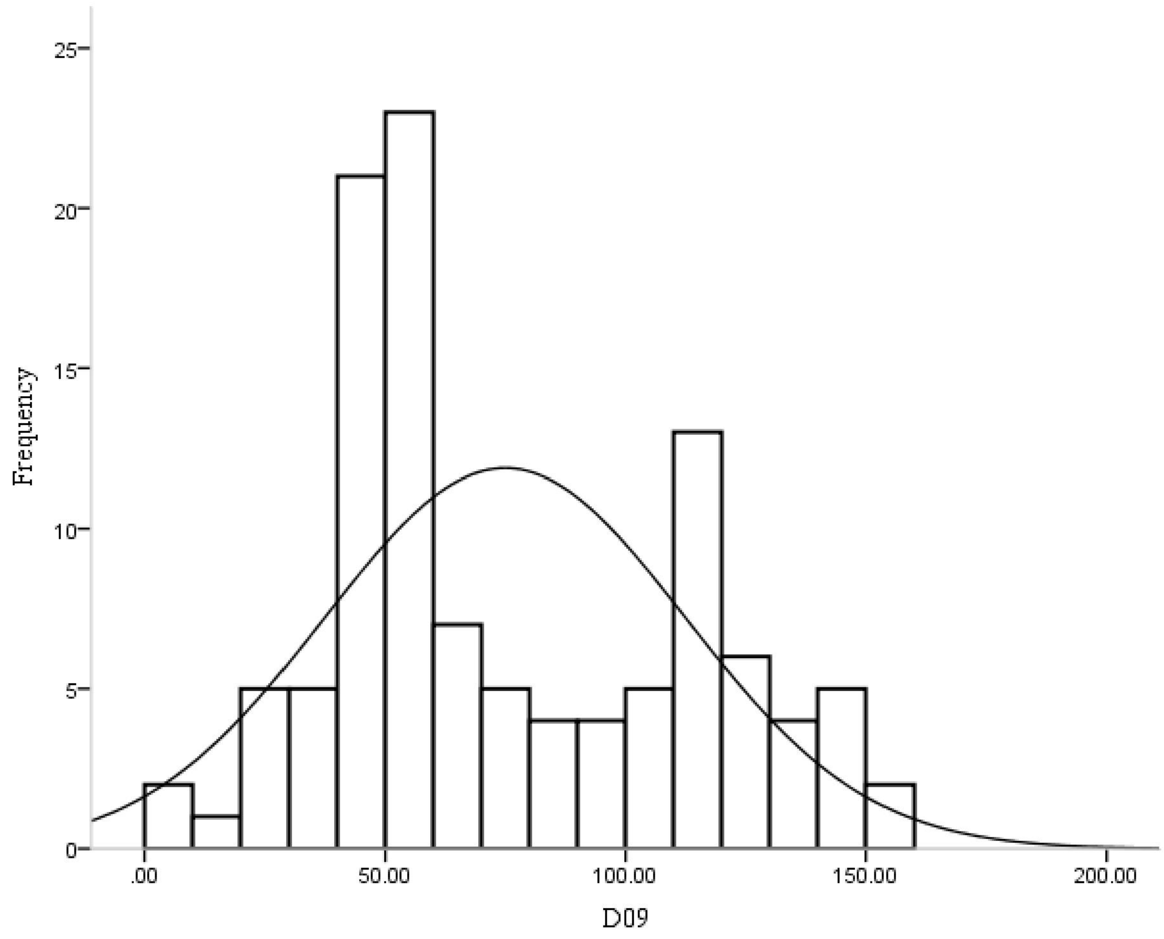

25
20
15
10
5
0
Frequency
.00
50.00
100.00
150.00
200.00
D09

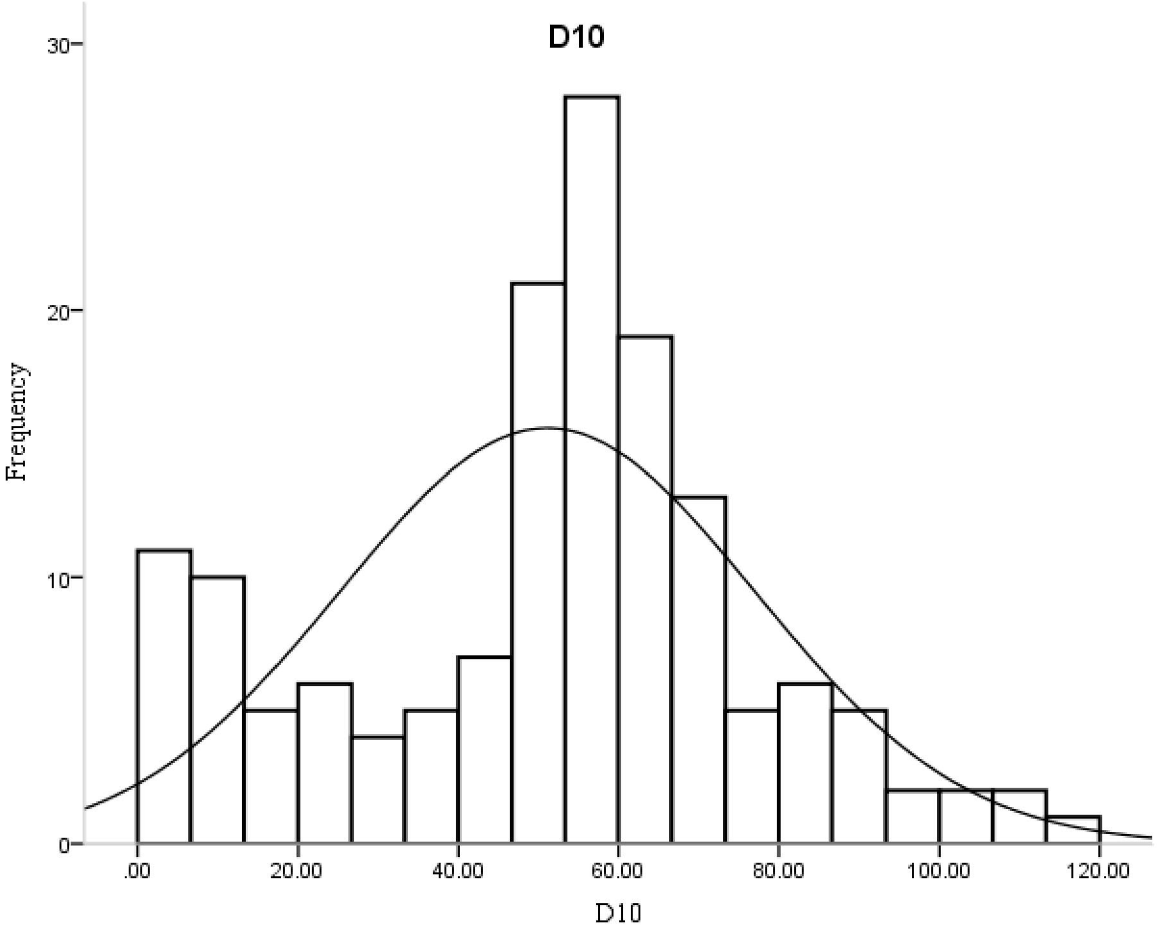
D10
30
20
10
0
Frequency
.00
20.00
40.00
60.00
80.00
100.00
120.00
D10

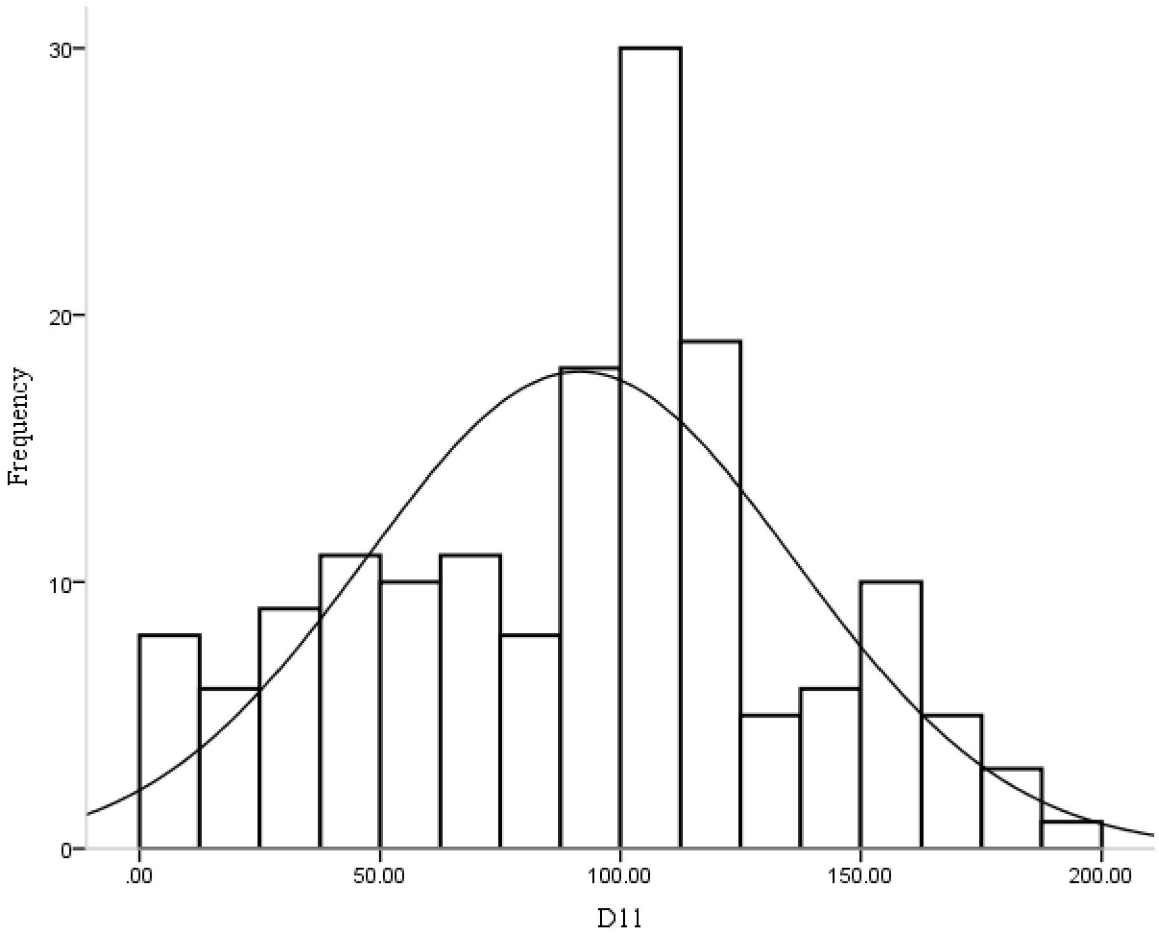

30
20
10
0
Frequency
.00
50.00
100.00
150.00
200.00
D11

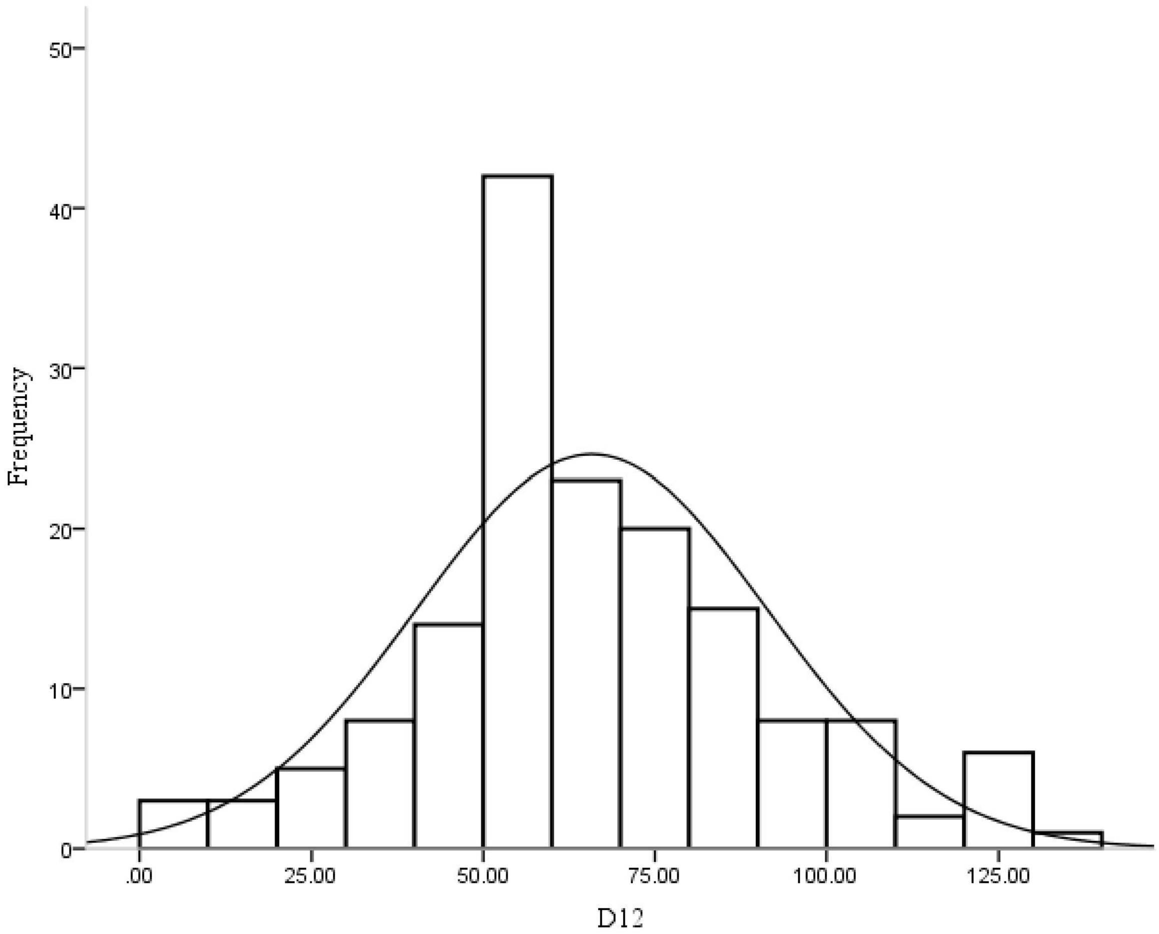

50
40
30
20
10
0
Frequency
.00
25.00
50.00
75.00
100.00
125.00
D12

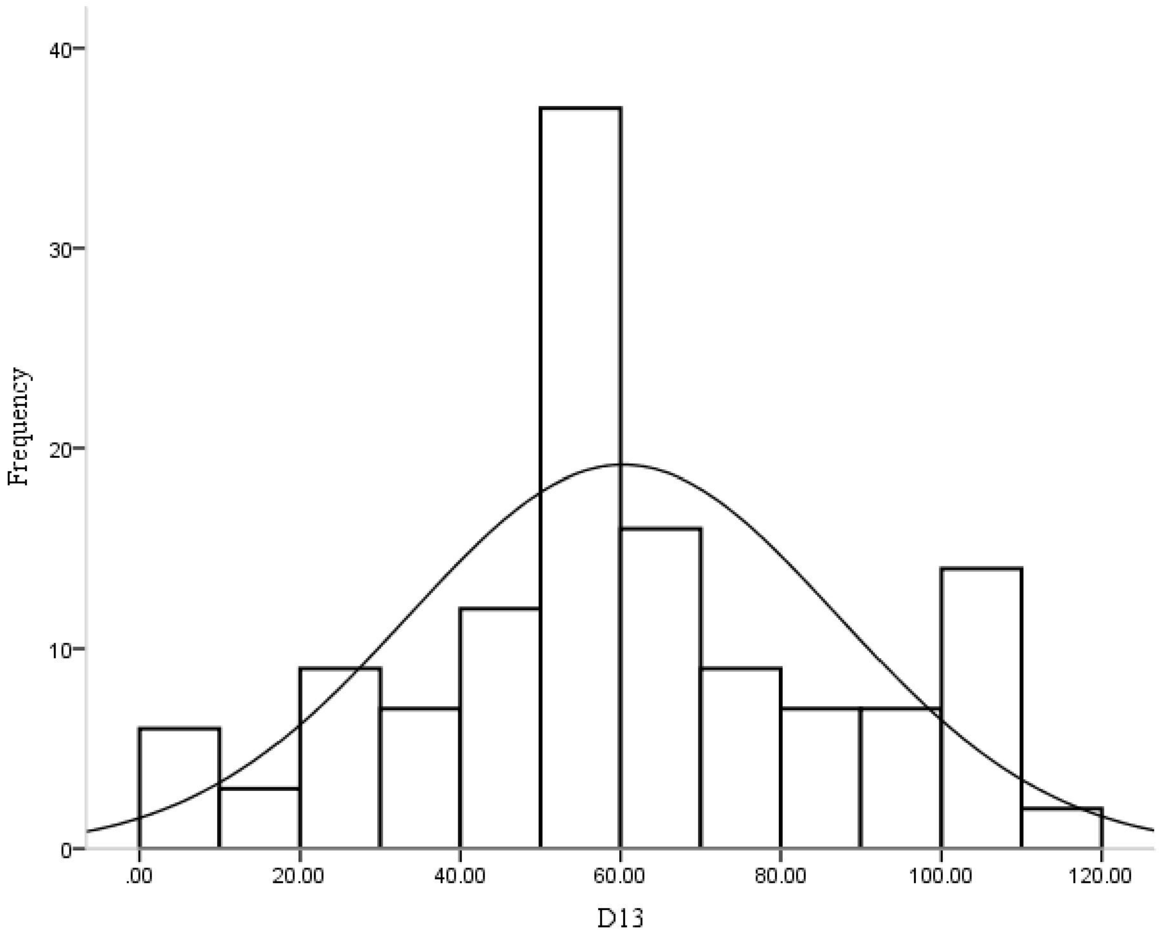

Figure S1 The frequency distribution of the polymorphic loci in each chromosome. The unit of the abscissa is centiMorgan (cM); the y-axis is percentage.
