# Supplementary material for: Toward allotetraploid cotton genome assembly: integration of a high-density molecular genetic linkage map with DNA sequence information
Source: BMC Genomics. 2012 Oct 9;13:539. doi: 10.1186/1471-2164-13-539 (PMC3557173; doi:10.1186/1471-2164-13-539)
Supplement: Additional file 6 — Figure S2. Functional annotation of 2,748 represented unigenes. [file 1471-2164-13-539-S6.doc]

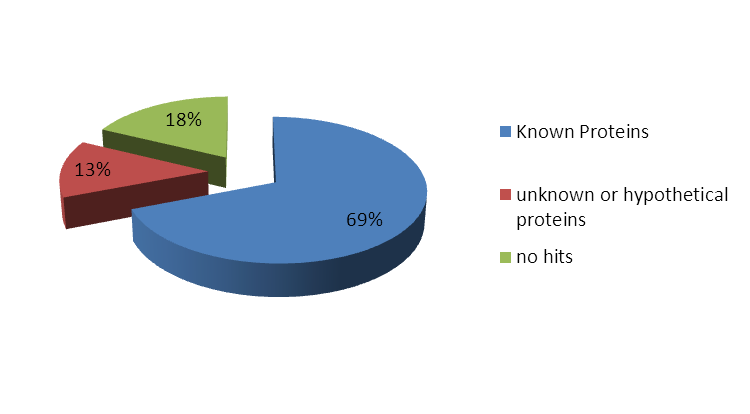


**Figure S2. Functional annotation of 2,748 represented unigenes with E-value ≤1e-5.**

Note: All candidate unigenes were subjected to homology searches against the NCBI RefSeq plant protein database (Release 53, May 10, 2012) using the Blastx alignment program.
