# Supplementary material for: Toward allotetraploid cotton genome assembly: integration of a high-density molecular genetic linkage map with DNA sequence information
Source: BMC Genomics. 2012 Oct 9;13:539. doi: 10.1186/1471-2164-13-539 (PMC3557173; doi:10.1186/1471-2164-13-539)
Supplement: Additional file 7 — Figure S3. Descriptive statistical analysis of the Blast alignment results against the NCBI RefSeq plant database. [file 1471-2164-13-539-S7.doc]

**
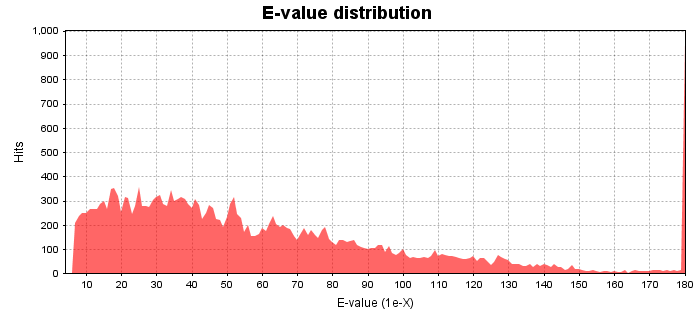

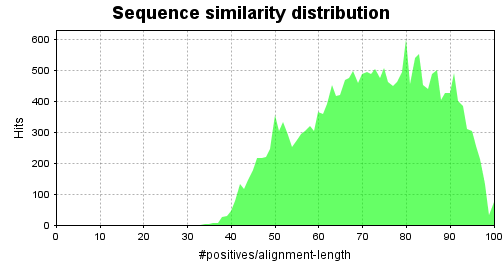
**

**B**

**A**

**
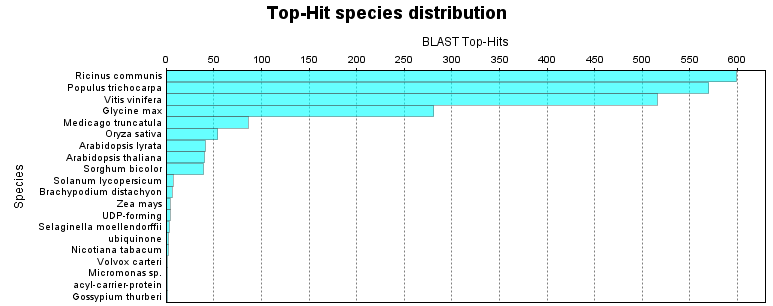
**

**C**

**Figure S3. Descriptive statistics charts for Blast alignment results against the NCBI RefSeq plant database.**

Note: A) E-value distribution for the BLAST hits. B) Similarity distribution for the best BLAST hit for each sequence. C) Top-Hit Species distribution: number of times each species appears in the BLAST hit results.
