# Supplementary material for: Toward allotetraploid cotton genome assembly: integration of a high-density molecular genetic linkage map with DNA sequence information
Source: BMC Genomics. 2012 Oct 9;13:539. doi: 10.1186/1471-2164-13-539 (PMC3557173; doi:10.1186/1471-2164-13-539)
Supplement: Additional file 9 — Figure S4. Functional classification of the 2,748 unigenes that were assigned GO terms. [file 1471-2164-13-539-S9.doc]

**B**

A


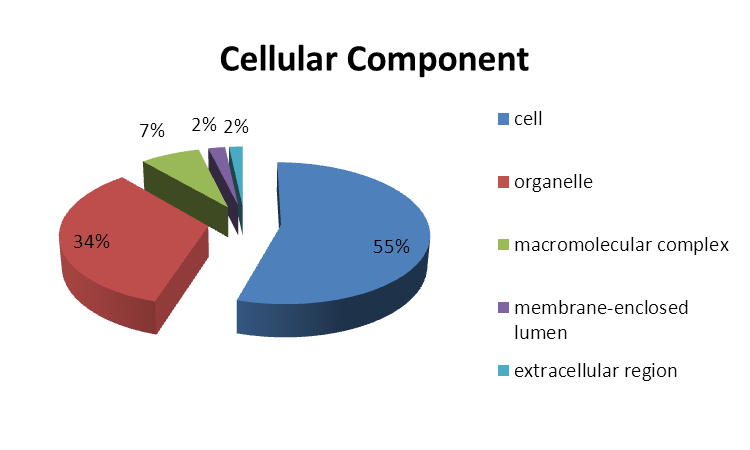


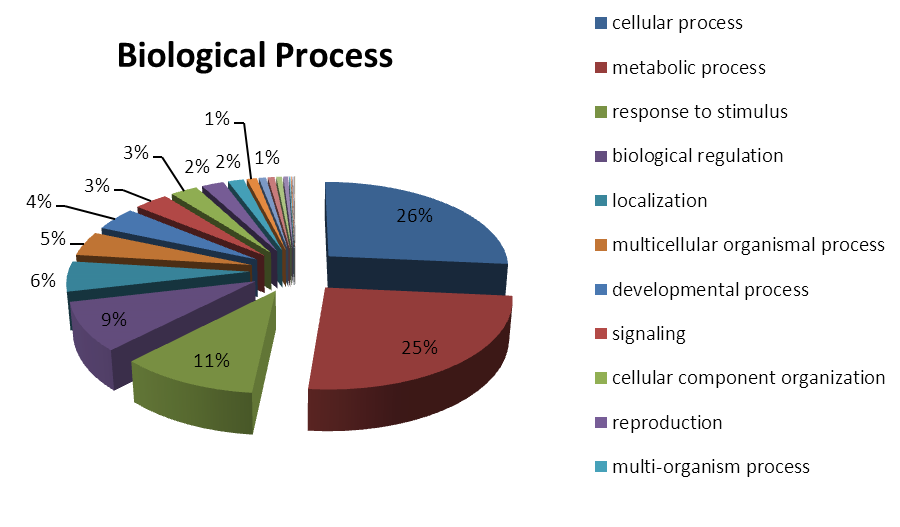

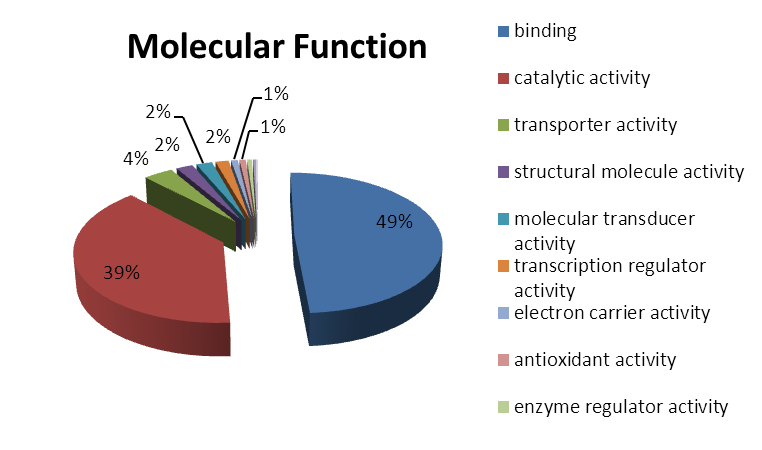


C

B

**Figure S4. Functional classifications for 2,748 unigenes assigned with GO terms.**

Note: A) Cellular component. B) Biological process. C) Molecular function. More detailed information is provided in additional file 8.
