# Supplementary material for: Toward allotetraploid cotton genome assembly: integration of a high-density molecular genetic linkage map with DNA sequence information
Source: BMC Genomics. 2012 Oct 9;13:539. doi: 10.1186/1471-2164-13-539 (PMC3557173; doi:10.1186/1471-2164-13-539)
Supplement: Additional file 11 — Figure S5. Functional classification of the 2,748 unigenes that were assigned level 2 KEGG metabolism terms. [file 1471-2164-13-539-S11.doc]

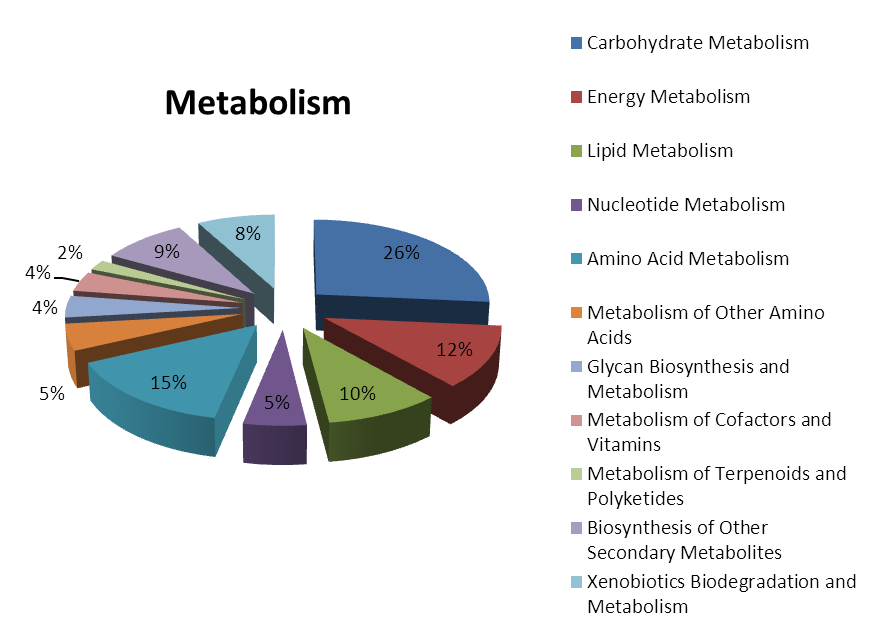


**Figure S6. Functional classifications for 2,748 unigenes assigned with KEGG Metabolism terms (level 2).**

Note: More detailed information is provided in additional file 10.
